# FUCCItrack: An all-in-one software for single cell tracking and cell cycle analysis

**DOI:** 10.1371/journal.pone.0268297

**Published:** 2022-07-06

**Authors:** Hubert M. Taïeb, Luca Bertinetti, Tom Robinson, Amaia Cipitria

**Affiliations:** 1 Department of Biomaterials, Max Planck Institute of Colloids and Interfaces, Potsdam, Germany; 2 B CUBE Center for Molecular Bioengineering, TU Dresden, Dresden, Germany; 3 Department of Theory and Bio-Systems, Max Planck Institute of Colloids and Interfaces, Potsdam, Germany; 4 Biodonostia Health Research Institute, Group of Bioengineering in Regeneration and Cancer, San Sebastian, Spain; 5 IKERBASQUE, Basque Foundation for Science, Bilbao, Spain; University of Bayreuth: Universitat Bayreuth, GERMANY

## Abstract

Beyond the more conventional single-cell segmentation and tracking, single-cell cycle dynamics is gaining a growing interest in the field of cell biology. Thanks to sophisticated systems, such as the fluorescent ubiquitination-based cell cycle indicator (FUCCI), it is now possible to study cell proliferation, migration, changes in nuclear morphology and single cell cycle dynamics, quantitatively and in real time. In this work, we introduce FUCCItrack, an all-in-one, semi-automated software to segment, track and visualize FUCCI modified cell lines. A user-friendly complete graphical user interface is presented to record and quantitatively analyze both collective cell proliferation as well as single cell information, including migration and changes in nuclear or cell morphology as a function of cell cycle. To enable full control over the analysis, FUCCItrack also contains features for identification of errors and manual corrections.

## Introduction

Cell cycle dynamics and migration are essential processes that govern many critical mechanisms of life, from embryonic development to adulthood, as well as pathological alterations, such as tumor formation and progression. However, most cell migration analyses are typically done blind with respect to the cell cycle status. Only in the last decade, several cell cycle reporters such as the fluorescent ubiquitination-based cell cycle indicator (FUCCI) system have emerged [1], which allows imaging of cell cycle dynamics by visualizing individual G1 and S/G2/M phases both *in vitro* and *in vivo*, in real time and without exogenous dyes [2–4]. This enables a wide range of studies aimed at understanding cell behavior in more depth such as cell deformability and proliferation as a function of cell cycle dynamics [5] or tumor spheroid growth within compliant vs. stiff hydrogels [6]. Additionally, mathematical models of FUCCI systems are created to relate cell migration [7] or tumor spheroid formation [8] with cell cycle dynamics. The original FUCCI probe used mKO2-hCdt1 (30/120) for the G1 phase and mAG-hGeminin (1/110) for the S/G2/M phase [1]. This was later improved with the FUCCI2 cell cycle reporter, which employed mCherry-hCdt1 (30/120) for G1 and mVenus-hGeminin (1/110) for S/G2/M phase [9]. FUCCI2 offers enhanced contrast and the possibility to spectrally distinguish the signals from the widely used fluorophore GFP for applications such as parallel monitoring of protein subcellular localization and/or signaling events [9]. However, FUCCI or FUCCI2 cannot be used to separate the cells in the G0 phase from those in G1 since Cdt1 is thought to be expressed in both phases [1]. This led to the development of new methods adding to the original FUCCI system whereby cells in the G0 phase were separated from those in G1. This construct used mVenus-p27K^-^ for G0, mCherry-hCdt1 (30/120) for G1 and AmCyan-hGem (1/110) for S/G2/M [10]. Other groups also developed FUCCI4, a set of four fluorescence indicators that could resolve independently every single phase of the cell cycle (G1, S, G2 and M) [11], which traditional FUCCI or FUCCI2 could not separate. To the best of our knowledge, FUCCI is the most useful and complete tool to visualize all phases of the cell cycle dynamics in real time [12]. Our current work is focused on FUCCI and FUCCI2 but could be expandable to any two-color cell cycle reporter.

Conventional cell tracking used to be a manual task [13], with users needing to select each cell at a time, frame by frame. This approach was inherently time consuming, especially when looking at long time-lapse imaging, and prone to different bias, due to subjective definition of the center of the cell or user inconsistencies. More recently, many platforms proposed automatic single-cell tracking with different technical solutions [14], both from an open-source initiative on FIJI (ImageJ) or other platforms [15–17], or from proprietary software. These tools are useful to track cell migration, however, some cannot handle cell division in a consistent, user-independent manner. This may result in wrong tracking of daughter cells, without the possibility to inspect, edit or adjust the tracking.

This is where the FUCCI cell cycle reporter has an extraordinary benefit with respect to simpler live staining. During G1, only the fluorophore mCherry-Cdt1 (30/120) is expressed. Expression of the fluorophore mVenus-hGeminin (10/110) inside the nucleus indicates the beginning of the S/G2/M phase. Thus, by expressing first the fluorophore mCherry then mVenus, the FUCCI system provides a defined pattern to track single cells and stop the tracking when the intensity for mVenus drops due to the cell division. This not only allows to monitor the cell cycle dynamics, but also tracking cell migration parameters such as trajectory and velocity, as a function of cell cycle state, in a single setup and in real time [18]. Paradoxically, this benefit can also turn into a disadvantage, since it means that the fluorophore used for tracking does not have a constant intensity over time for each cell. Alternating fluorescence channels (mCherry/mVenus) with an inherent intensity variability makes traditional automatic image processing more complicated to implement.

To the best of our knowledge, only three software have been reported to treat FUCCI time-lapse datasets in an automatic or semi-automatic way [19–21]. One is not publicly available [21], one requires multiples applications to be run (Fiji and Columbus) [20], and the latest one was released recently on MATLAB [19]. The latter can perform migration analysis and includes a FUCCI plug-in to extract nuclear intensity and obtain cell cycle duration. However, the software is focused on tracking cell migration and does not perform any segmentation of the nucleus or cell shape. It also does not use the full potential of the FUCCI system to automatically detect cell division since the tracking is independent from the FUCCI intensity. In addition, it is impossible to extract collective cell proliferation parameters such as number of cells over time and remains targeted to single cell behavior only.

Here, we introduce FUCCItrack, a MATLAB based open-source application that allows non-programming specialists the possibility to perform automated, as well as supervised tracking and segmentation of multitude of fluorescent cell lines. Although tailored for FUCCI2, the software can also work with any other two-color fluorophore combination. With minimum modification, it is also possible to use it for only one fluorescence channel, such as fluorescently labeled cells or any fluorescent object for particle image velocimetry analysis (fluorescent beads). It can be used as a robust quantitative tool in a vast range of two-dimensional (2D) experimental setups. It includes a user-friendly visualization module to plot the results in a graphical format directly within the application and export the graphs or movies. FUCCItrack allows users to perform rapid and efficient automatic nucleus segmentation, as well as cell shape segmentation if an additional fluorophore is used, to then investigate cell cycle dynamics, proliferation, migration and morphology as a function of time and cell cycle state.

## Materials and methods

### Cell culture

Human metastatic breast cancer cells MDA-MB-231 [22] (ATCC, #HTB-26) and MCF7 [23] (#HTB-22) were genetically modified with the FUCCI2 reporter (MDA-FUCCI2, MCF7-FUCCI2) as described previously [18]. They were cultured following standard methods. Briefly, low glucose Dulbecco’s modified eagle’s medium (Sigma, #D6046) was supplemented with 1% penicillin/streptomycin (Gibco, #1514–122, 10^4^ units/mL of penicillin and 10 mg/mL of streptomycin) with 10% v/v fetal bovine serum superior (Sigma, #S0615). The cells were grown at 37°C with 5% CO_2_ on regular Nunc^TM^ 100x17 mm petri dishes (Thermo Fischer, #150350) for regular passages.

### Time-lapse FUCCI2 experiment

Prior to imaging, MDA-FUCCI2 cells were washed with phosphate buffered saline (PBS), detached with trypsin (PAN-Biotech, #38220000) and centrifuged at 300 g for 5 minutes. They were then seeded at 7500 cells/mL (200 μL) on a 96-glass bottom microplate (Greiner Bio-one, #655892) and allowed to adhere for 24 hours before imaging. The cell seeding density was chosen so that the wells remained subconfluent until the end of the imaging period and thereby reduce possible errors during single cell tracking. Cells were then placed in a stage top incubator at 37°C and 5% CO_2_ (Okolab, UNO-T-H-CO2) mounted on an inverted epifluorescence microscope (Zeiss, AxioObserver 7) for long term time-lapse imaging.

### Cell actin staining

Some experiments were performed using the SiR-Actin Kit (Spirochrome, #SC-001) which is a live F-actin cell staining. Briefly, cells were incubated in suspension for 6 hours with the cell culture media containing the SiR-Actin at a concentration of 100 nMol/L. Cells were then seeded as described in the previous section, with the media containing the SiR-Actin to allow a consistent F-actin staining over time and throughout the different cell generations.

### Image acquisition

All images were acquired with a Zeiss AxioObserver 7 inverted epifluorescence microscope. The objective was a 10x, 0.45 numerical aperture (Zeiss, #420641-9910-000). For each well of the microplate one field of view was taken. Both fluorescence channels mVenus and mCherry were recorded with 100% LED intensity at 511 nm and 555 nm illumination wavelength, respectively. For the experiments with SiR-Actin, 100% LED intensity at 630 nm was used in addition. The filter sets used were Zeiss #46 HE (500/25 nm excitation and 535/30 nm emission) for mVenus, Zeiss #45 (560/40 nm excitation and 630/75 nm emission) for mCherry and Zeiss #50 (640/30 nm excitation and 690/50 nm emission) for SiR-Actin. All channels were recorded at 300 ms exposure. Images were acquired in intervals of 30 minutes for 90 hours.

### Single cell tracking

After seeding the cell of interest in the field of view, information about the segmented nucleus in both fluorescence channels is recorded, including coordinates of center of mass, area and FUCCI2 intensities. To track the cells in the next frame, the software uses a localized search region around the previous position. The size of this search region can be changed by the user, depending on the specifics of the cell line and interval used for imaging. A local adaptative threshold method is used to segment both mCherry and mVenus fluorescence channels in this search region and compares the several segmented nuclei found (coming from the cell of interest or others nearby) with the one in the previous frame in term of distance, fluorescence intensity, and shape. The algorithm then picks between the several segmented nuclei the candidate that is most similar to the previous time point. If the algorithm fails to find the corresponding nuclei from one frame to the other, it is possible to use the “Manual centering” option to manually correct the cell tracking, while the rest of the segmentation proceeds automatically.

We tested our tracking method with a publicly available FUCCI2 dataset (https://doi.org/10.5281/zenodo.4179316) and could not measure any significant differences with CellMAPtracer [19] (S1 Fig).

### Segmentation of FUCCI2 time-lapse

For every single cell, the entire field of view was cropped around the cell of interest thanks to the tracking method, which results in the center of the cropped image to be the center of the nucleus of interest. On this image, FUCCItrack performs a binarization using an adaptive threshold based on a mean local intensity to account for nuclei of different intensities potentially presents in the region of interest. From the binary mask, all cluster of pixels smaller than 6 μm in diameter (this value can be modified by the user) were then filtered and removed. An additional step of filtering big clusters is performed for cluster representing more than 35% of the region of interest. Then, a morphological operation of closing (with a disk element of 1 pixel) is performed and all the holes in the clusters present in the binary image are filled. If several clusters are left in the region of interest, a last step is performed to only keep the cluster, which is closest to the center of the image, and the others are discarded. The result of the segmentation for one exemplary cell at one time point can be visualized on S2 Fig and during its whole cell cycle in S4 Movie. In addition, the segmentation results of all dividing cells can also be found in the following public repository (https://dx.doi.org/10.17617/3.7l)).

## FUCCItrack software description and workflow

### FUCCItrack software installation

FUCCItrack is built with MATLAB (R2019b, v 9.7) and is open-source, available as the (a) MATLAB App/Toolbox and (b) MATLAB source code or (c) a standalone application (MATLAB does not need to be installed to use the software). The application can be run on Windows, macOS or GNU/Linux operating systems.

- MATLAB App/Toolbox. This can be found at https://github.com/hubert-taieb/FUCCItrack. Users should download the “FUCCItrack.mlapinstall” file, open MATLAB, go to the APPS tab and click “Install App”. Select the downloaded file and then install it. It is possible to open it from the APPS tab or via the command line.
- MATLAB source code. Users should download the package “FUCCItrack_2021.02_source_code.rar” at https://github.com/hubert-taieb/FUCCItrack and extract the files. Then open MATLAB and go to the folder containing the package. Directly run “FUCCItrack_V2021_02.mlapp” within the MATLAB command line.
- Standalone application. The executable can be found at https://github.com/hubert-taieb/FUCCItrack under “FUCCItrack_installer.exe”. Directly open the file from the explorer and follow the instructions.

### Software description and workflow

**Fig 1:**
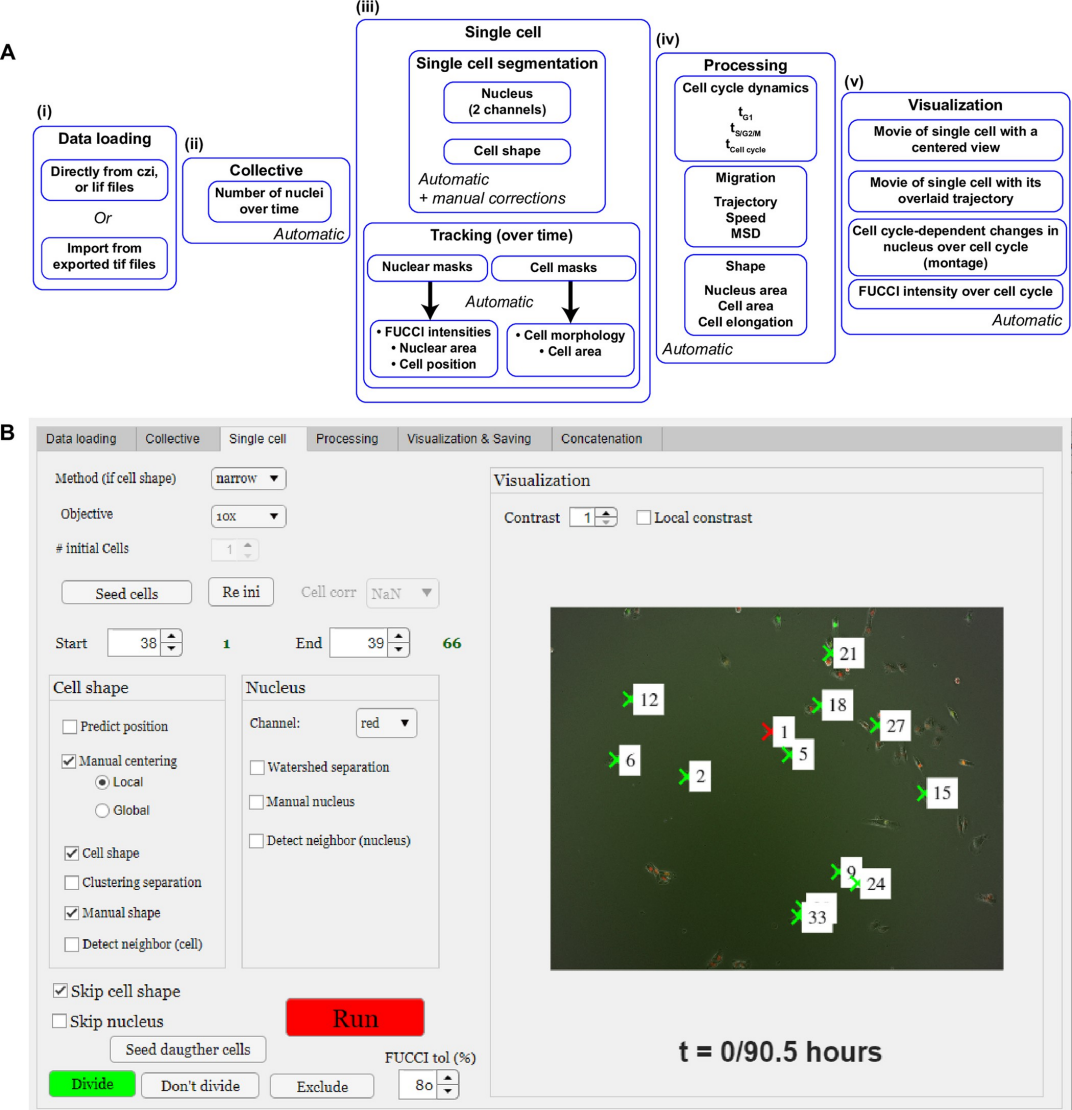
Workflow and user interface of FUCCItrack. (**A**) Workflow describing the use of FUCCItrack, from loading to visualization. (**B**) Single cell panel describing the segmentation and tracking user interface.

FUCCItrack is a graphical user interface (GUI) that is tailored for FUCCI2 system, but also extensible to other two-color fluorophore combinations, as described above. The complete workflow is described in Fig 1A and refers to the five main independent modules to analyze collective as well as single FUCCI2 cells. There is one additional concatenation module, which is optional and only described below. Some are fully automatic, and others are semi-automatic, with options for manual corrections. FUCCItrack also allows to save and reopen a session with the application returning to the previously saved state. See the FUCCItrack’s user guide (S1 File) for a complete user’s guide and description to FUCCItrack and how to use it.

#### Data loading module

Processing of the images begins by loading the native microscope data as described in Fig 1A(i) (Zeiss”.czi” or Leica “.lif” file). This allows to automatically extract the metadata, such as the spatial resolution and time interval, and the fluorescence/phase contrast channels used in the experiment. It also avoids having any data loss due to compression needed for precise quantification. If the native microscope data is not available, FUCCItrack can load multi-tiff stacks (the layers of the stacks representing different time intervals) or sequential-tiff images. For this, the user must specify manually the pixel size (in μm/pixel) and the time interval (in hours) and make sure to import the raw microscopy images without data compression. The preferred mode of import is to use the raw microscopy files (.czi or.lif) that use open-source OME bio-formats library [24] since it requires less user inputs and is more standardized.

For FUCCI2 datasets, the minimal requirement is to have two fluorescence channels for the nucleus (named mCherry and mVenus), and one for the cell itself (phase contrast image and/or SiR-Actin fluorescence for example). Epifluorescence 2D images were used for this study, but maximum projection of 3D confocal image stacks or individual slices also yields similar results.

Using these inputs, FUCCItrack then runs at two different levels: (i) collective and (ii) single cell level.

#### Collective module

The collective level allows users to extract the cell number as a function of time in a fully automated way (Fig 1A(ii)). Despite its simplicity, this counting method can already give valuable information about the cell proliferation under any experimental conditions, using 2D epifluorescence microscopy in real time.

FUCCItrack makes use of the fluorescence channels from the nucleus and automatically counts the number of cells as a function of time, using an adaptive thresholding approach based on a mean local intensity [25]. This adaptive thresholding is necessary for two reasons. In the FUCCI2 system, the fluorophores are attached to proteins that are getting degraded or produced over time. This means that two cells on the same field of view can have different intensity levels at the same time even though they are both in the same cell cycle phase. Moreover, the absolute mCherry and mVenus maximum intensities that a cell reaches during its cell cycle is not the same throughout a population of cells. For these reasons, it is not possible to use a global threshold approach with one cut-off values for the intensity. Instead, a locally automatically defined threshold is computed and yields the best segmentation results.

**Fig 2:**
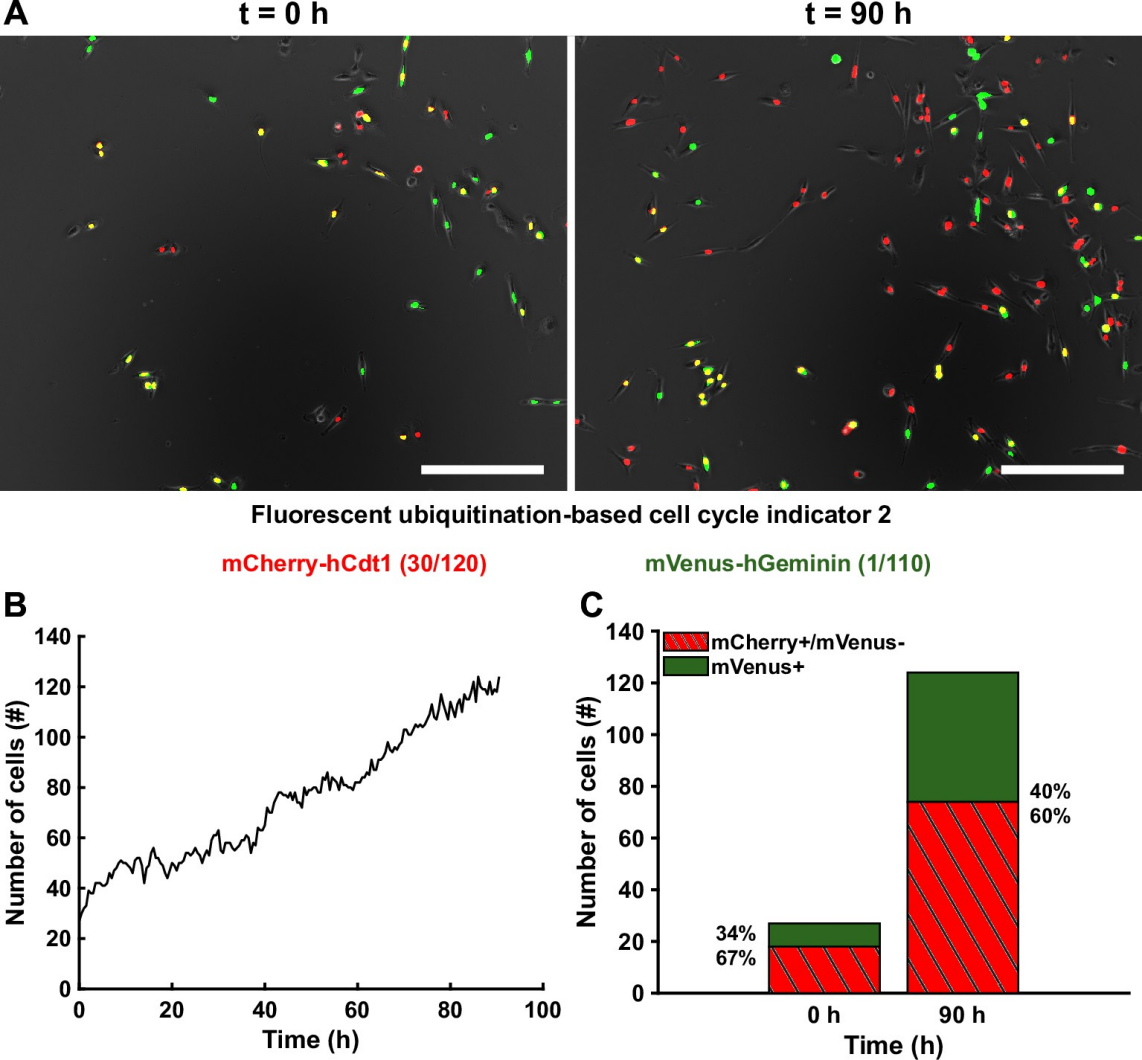
Cell proliferation of MDA-FUCCI2 human breast cancer cells at collective level. (**A**) Time-lapse of MDA-FUCCI2 over 90 hours of imaging. Scale bars are 200 μm. (**B**) Number of cells as a function of time obtained from the automatic collective segmentation. (**C**) Fraction of mCherry^+^/mVenus^-^ (G1 phase) and mVenus^+^ (S/G2/M phase) cells at the beginning and at the end of the 90 hours of imaging.

In addition, since the two fluorophores are cell cycle reporters, the collective level permits to gain insight on the fraction of mCherry^+^ and mVenus^+^ cells at a given time point based on the two fluorescence channels segmented. An extra-step to verify whether the same cell is present in both channels is then applied and cells are categorized as mCherry^+^/mVenus^-^ (G1 phase) or mVenus^+^ (S/G2/M phase). This module gives an overview of the cell proliferation behavior as well as fraction of cells in distinct G1 or S/G2/M cell cycle phase (Fig 2) but does not provide any tracking of single cell over time.

#### Single cell module

**Fig 3:**
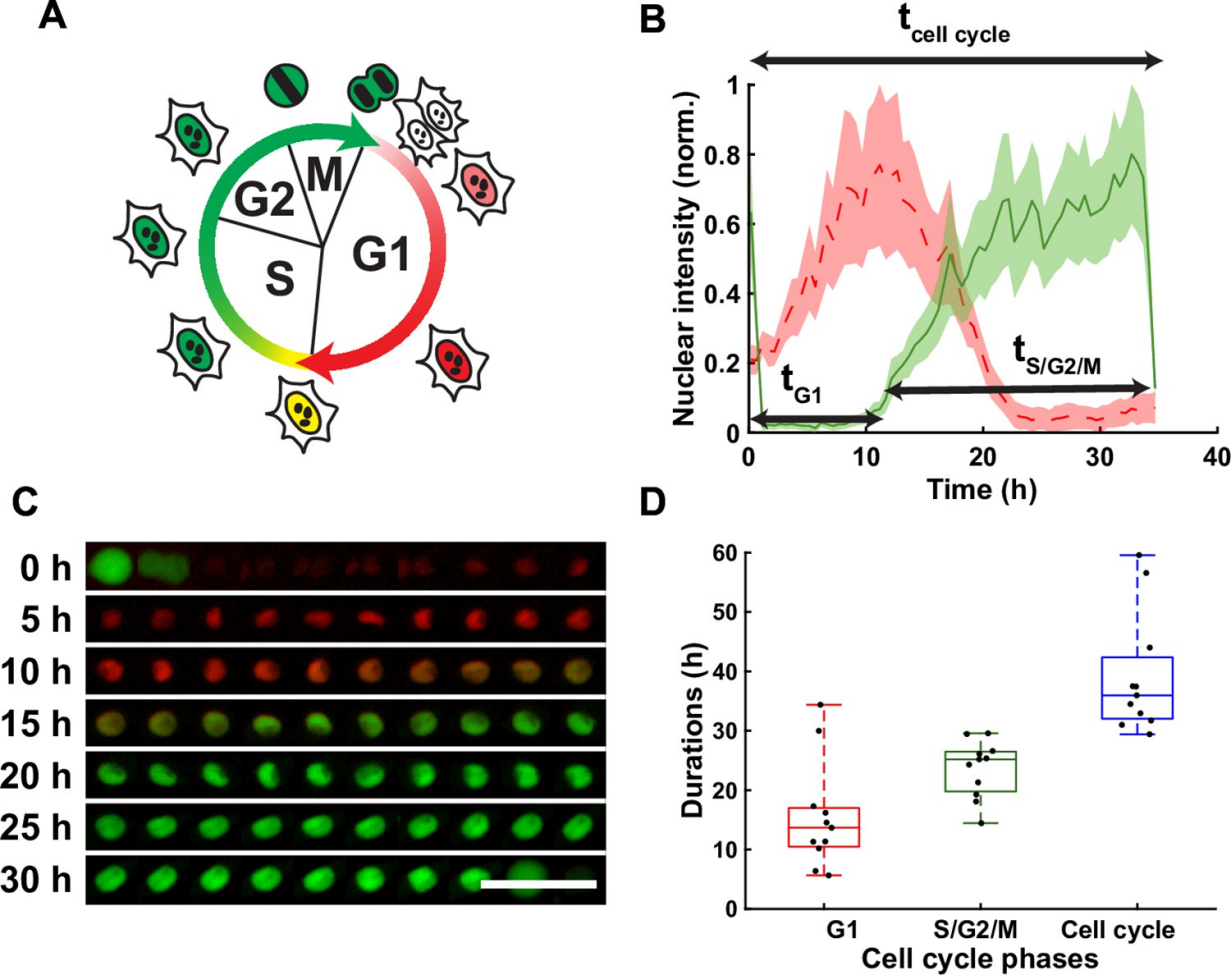
Single cell tracking with the FUCCI2 system. (**A**) FUCCI2 cartoon representing the mCherry (red) and mVenus (green) fluorescence of the nucleus during the cell cycle. (**B**) Normalized fluorescence intensity in mCherry (dashed red) and mVenus (green) for Cell #8, with the corresponding duration of the phases. The line is the average, and the shaded area is the standard deviation. (**C**) Corresponding fluorescence images of the segmented nucleus over time. Scale bar is 100 μm. (**D**) Duration in hours of the cell cycle phases: G1 (red), S/G2/M (green) and total cell cycle (blue). The FUCCI2 cartoons was adapted from Sakaue-Sawano et al. [1], Copyright (2008), with permission from Elsevier.

Automatic collective segmentation cannot track mitosis events efficiently. This module (Fig 1A(iii)) allows to track single cells and segment shapes of nuclei as well as cell shape automatically. The single cell level, in contrast, opens a new realm of information to study single dynamics, migration (trajectory and velocity), and changes in nuclear and cell morphology throughout several generations of cells (Figs 3 and 5).

The tracking module is presented in Fig 1B. The user is invited to select a cell of interest manually (“Seed cells” button) to specify which cell from the field of view should be tracked. Starting from the current frame and after pressing “Run”, the automatic segmentation and tracking of this cell over time starts (Fig 1A(iii) and 1B).

As presented in Fig 1B and described more in detail in the user guide (S1 File), the user can run segmentation tasks on the datasets using two different panels. One for cell shape (if a live cell actin or cell membrane staining is used such as SiR-Actin), and one for the nucleus. If users have a live cell actin or cell membrane staining or analog, they should select the “Cell shape” to perform cell shape segmentation. Also, when using the option to detect already segmented neighbor cells, the tracking accuracy improves with the number of already segmented cells. To register cell division, FUCCItrack uses the precise and characteristic pattern upon division. Namely, cells will have an intense mVenus signal inside the nucleus before dividing (Fig 3B), which will decrease after division. The tracking algorithm exploits this behavior and monitors the mVenus signal coming from the region of interest before and after cell division. When the mVenus signal is found to be much weaker in the frame after division, FUCCItrack will trigger a halt in the tracking with a message in the status panel. If the division of the original manually seeded cell indeed occurred, the user is invited to register cell division by using the “Divide” button, followed by the “Seed daughter cells”. This automatically seeds the two daughter cells for further tracking and create the lineage between cell generations. Users can choose to perform automatic cell shape segmentation if a specific fluorescence dye is present in their experiment or manual segmentation using the phase contrast/bright field images. This remains optional, and users can also use FUCCItrack to only perform tracking and segmentation of nuclei.

#### Processing module

The processing module allows users to extract the information from cell tracking and segmentation to output cell cycle dynamics, migration parameters and shape analysis.

Once the segmentation is performed, either automatically or with manual corrections, the intensity values inside the nuclear mask of each channel are extracted. The mean intensity inside the nucleus is computed to determine the duration of the cell cycle phases in the “Processing” tab (Fig 1A(iv)). The intensity curves are normalized by the maximum intensity reached during its cell cycle, in both mCherry and mVenus channels independently, due to the variability between cells and fluorescence channels. From these curves the durations of the cell cycle phases can be obtained (Fig 3A and 3B). The beginning of the S/G2/M phase is then defined as the time when the intensity of mVenus exceeds 5% (this value can be modified by the user depending on their cell lines) of the maximum intensity. The same method applies for the beginning of the G1 phase (Fig 3B). The software computes the cell cycle dynamics parameters (t_G1_, t_S/G2/M_; t_Cell cycle_) for each single cell tracked and the result can be exported in a text file.

**Fig 4:**
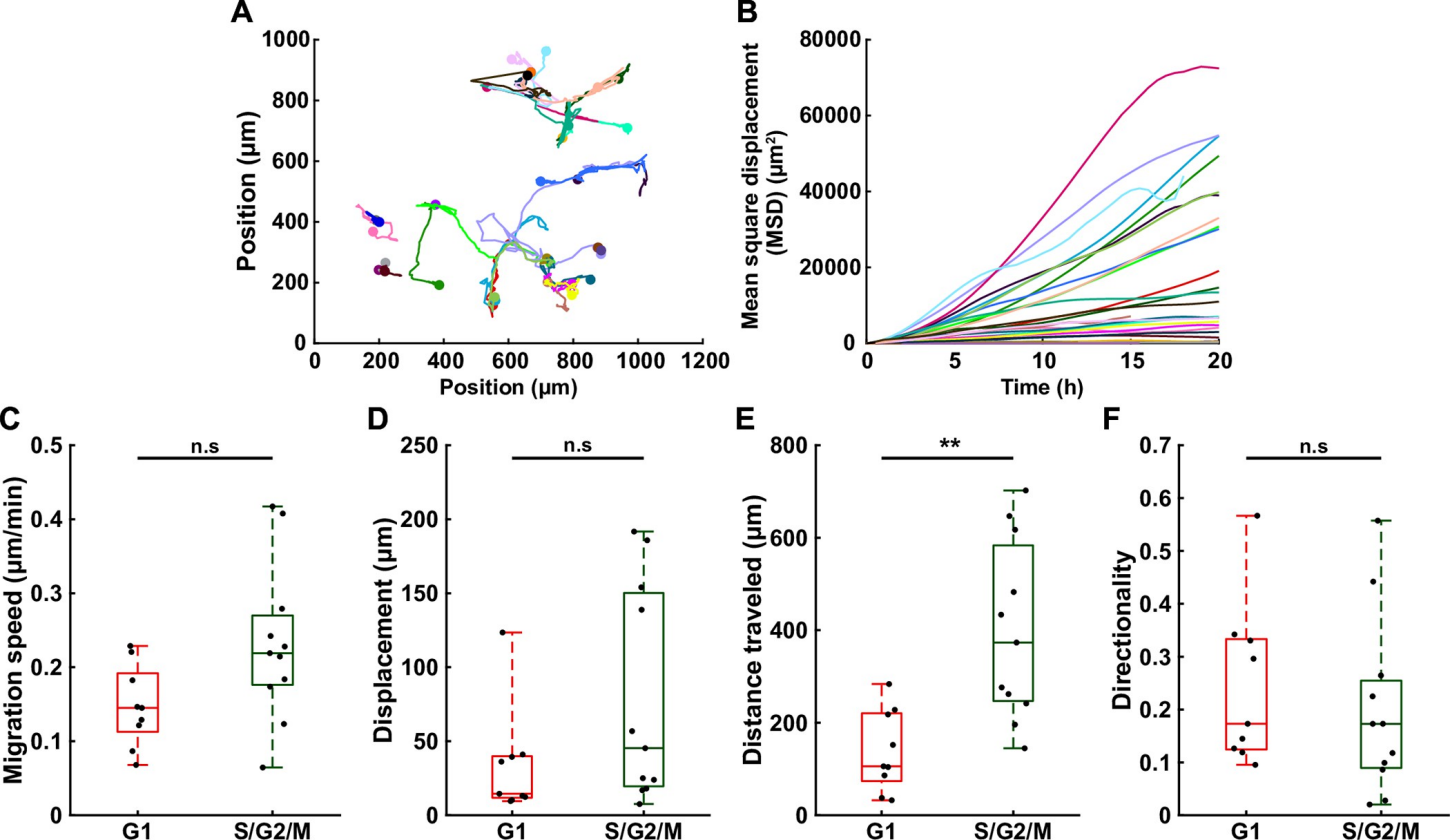
Cell migration analysis correlated with cell cycle state using the FUCCI2 system. (**A**) Trajectories of MDA-FUCCI2 cells during their cell cycle. (**B**) Corresponding mean square displacement (MSD) for every cell tracked (n = 24 cells). (**C-F**) Boxplots showing the difference between cell cycle state of MDA-FUCCI2 daughter cells (n = 11 cells) according to 4 migration features: (**C**) migration speed, (**D**) displacement (distance from beginning to end of trajectory), (**E**) total distance (total trajectory length) and (**F**) directionality (ratio displacement to total distance). Statistical analysis with respect to the control using a two-tailed Wilcoxon rank sum test, n.s: p > 0.05, *: p < 0.05, **: p < 0.01 and ***: p < 0.001.

The information about the position over time of every cell tracked is also available from the segmentation data for further cell migration-related analysis (Fig 4). Indeed, after segmentation, the position of each nucleus is determined by computing the center of mass of the resulting mask. Basic trajectory analysis can be computed automatically by pressing the “Add tracks” button and include cell migration speed, displacement, total distance traveled, directionality and mean square displacement (using the package msdanalyzer [26]). When FUCCI2 datasets are used, it is possible to extract these migration related parameters as a function of the cell cycle. All the results can be exported in a text file.

**Fig 5:**
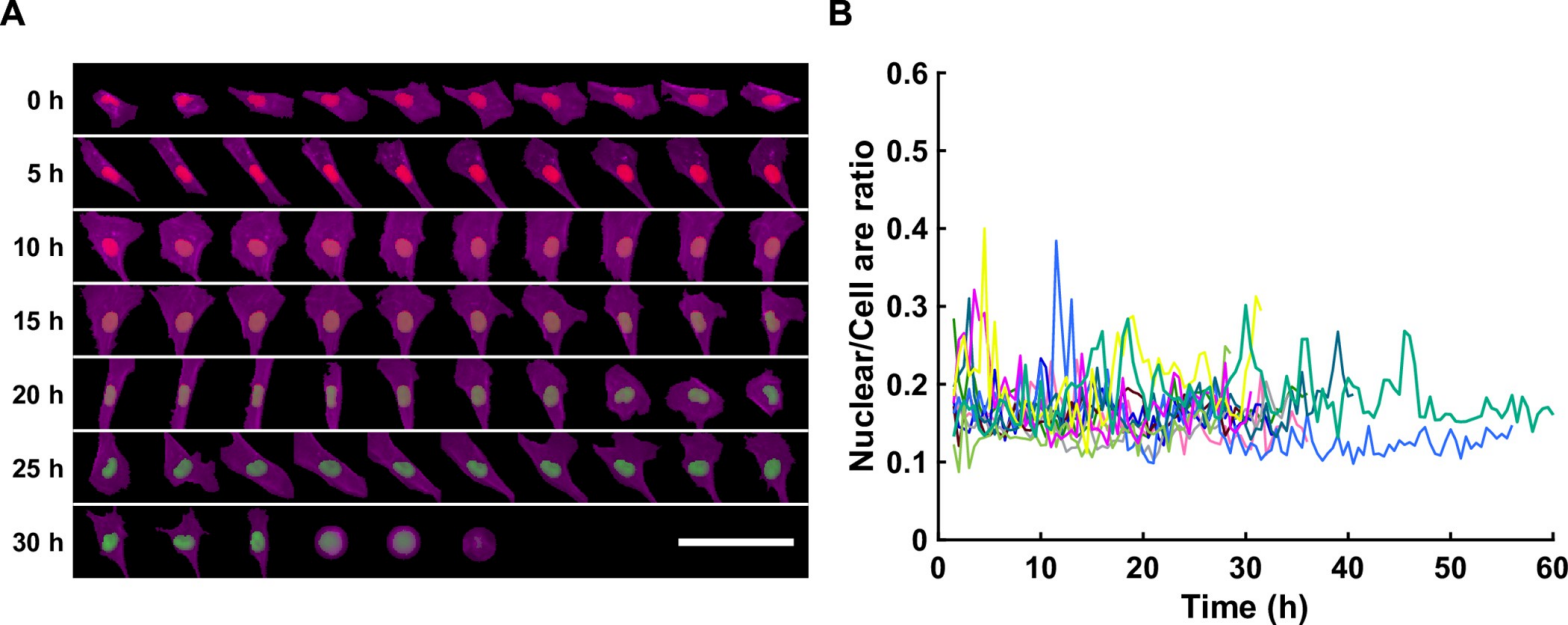
Cell and nuclear shape analysis. (**A**) Overlay of nuclei (red/green) and cell F-actin (magenta) as a function of time for and exemplary cell. Scale bar is 100 μm. (**B**) Ratio of nuclear area over cell area as a function of time for dividing cells (n = 11 cells).

Finally, because of the segmentation data, cell and nuclear morphological information can be used in correlation with cell cycle phases. The processing module performs morphological analyses of those data and calculates nuclear and cell area, equivalent diameter and elongation over time (Fig 5). For this application, only daughter cells are used, and the area is normalized by the area at two hours after division (this can be adjusted by users). Frames corresponding to two hours before the next division are excluded from the analysis, since they usually represent cell rounding up before division.

#### Visualization & saving module

In addition to the processing module, FUCCItrack also contains a dedicated module for automatic data visualization (Fig 1A(v)). All figures presented in this manuscript have been created automatically directly within the software (Figs 2B, 2C, 3B–3D, 4A–4F, 5A and 5B) and exported as vector and pixel-based files for a maximum quality.

More functions allow users to create movies automatically, showing only the region where the cell migrated over time, with its trace overlaid on the microscope images (S2 Movie), or a movie centered on the cell over time (S3 Movie).

#### Concatenation module

The description above relates to one time-lapse cell imaging experiment contained in one file. However, a concatenation module is also available. This module allows users to first run the application with a file from one experiment and then append another file that corresponds to the follow-up of the last time point of the previous experiment. This allows users to change environmental conditions (by even removing the plate from the microscope to change conditions) and still be able to match the cells in both experiments by correlating their position, thereby capturing changes in cell behavior under a dynamic environment.

## Results

### Collective level

Here we demonstrate the functionality of FUCCItrack by tracking, segmenting and analyzing MDA-FUCCI2 triple-negative breast cancer cells but the software could be used with other cell lines such as MCF7-FUCCI2 (S4 Movie). With the automatic collective module, the cell proliferation or the number of cells as a function of time is evaluated (Fig 2A and 2B). Thirty initial cells in the field of view proliferate to 120 cells at the end of the experiment, after 90 hours. Because the counting method can be applied to mCherry and mVenus channel independently, the user can evaluate the number of cells that express only mCherry (mCherry^+^/mVenus^-^, defined as G1 cells) or mVenus (mVenus^+^, defined as S/G2/M cells) as demonstrated in Fig 2C. Initially and after 90 hours, the ratio of cells in G1 and S/G2/M remains roughly constant at 60% and 40% respectively, indicating a normal proliferation. The results are obtained completely automatically, which makes this software a powerful tool to quantify cell proliferation easily and efficiently in real time.

### Cell cycle dynamics

A total of 11 cells were randomly selected for single cell tracking at the beginning of the experiment (Fig 1B). The user can choose to exclude cells due to fluorescence intensity problems (fluorescence intensity missing for mCherry or mVenus) or cells leaving the field of view during the tracking. The 11 selected cells and their descendants were tracked during the 90 hours of live imaging. An illustrative example with cell #8 is given in Fig 3B and 3C. Users can display the average fluorescence intensity inside the segmented nucleus for both mCherry (red line) and mVenus (green line), with the shaded red and green areas corresponding to the standard deviation inside the nucleus. From the total of 35 cells that were tracked, the median of the G1 phase was 14 hours, the median of the S/G2/M phase was 25 hours, and the total cell cycle duration median was 36 hours (Fig 3D).

### Migration analysis

From MDA-FUCCI2 tracked cells, cell migration related parameters can be computed throughout their cell cycle. First, the trajectories of all tracked cells are displayed in Fig 4A exhibiting a random cell migration. The mean square displacement (MSD) is evaluated for every cell and shown in Fig 4B, showing the heterogeneity in cell displacement. For the daughter cells, the correlation between the migration parameters and the cell cycle state is possible. Interestingly, there are no significant differences between migration speed (Fig 4C), displacement (Fig 4D) or directionality (Fig 4F) between the G1 and the S/G2/M phase. However, the distance traveled (Fig 4E) was significantly higher in S/G2/M with respect to the G1 phase, since it is measured as a summation of movement over time and the median time spent in S/G2/M phase is 25 hours instead of 14 hours for G1. Taken together, these findings indicate no change in migration behavior depending on the cell cycle state of MDA-FUCCI2.

### Shape analysis

Thanks to the FUCCI2 channels, we could segment the nucleus, and with the SiR-Actin channel we could determine the cell shape as projected area throughout the cell cycle progression (Fig 5A, S2 Fig, S4 Movie). For all cells, the ratio between nuclear to cytoplasm projected area remained constant around 0.2 (Fig 5B). The individual curves representing the evolution of each cell in terms of cell and nuclear area are also shown in the S3 Fig. In these figures, the nuclear and cell projected area was normalized to the first frame 2 hours after division of the parent cell so that only the increase in area during the cell cycle is presented.

Notably, we performed shape analysis of the nucleus of a MCF7-FUCCI2 cell and could observe a smaller variation in the projected area over time with respect to MDA-FUCCI2 (S4 Fig). Interestingly, MDA-MB-231 cells have been shown to move vertically or move above and below each other, whereas MCF7-FUCCI2 remain at the same height during their cell cycle progression [27]. This vertical movement of MDA-FUCCI2 can result in out of focus nucleus, resulting ultimately in a higher variation in the measurement of the nuclear area.

## Conclusion

Here we report FUCCItrack, an application that can be installed within MATLAB or as a standalone application and offers an all-in-one platform to perform segmentation of cell and nucleus, tracking, processing and visualization of cell cycle dynamics, along with migration and shape analyses. This can prove very useful to investigate cell cycle dynamics under different conditions such as osmotic stress for example. It provides non programming users a reliable and quantitative solution with a minimum effort as most processes are automatized. The gathering of all these tools into a common robust application diminishes the workload in comparison to more common manual tracking methods. We added the capability to generate graphics and movies from within the software directly. In addition, all results can be exported as text files to be further processed or analyzed by other tool. FUCCItrack is modular in its construction, which will facilitate future upgrades to include more features such as 3D cell tracking and segmentation.

## Supporting information

S1 File FUCCItrack user’s guide. (PDF)

S1 Fig Tracking accuracy of FUCCItrack compared to CellMAPtracer [19]. (**A**) Tracking results over the “FUCCI-2channels” datasets. The image contains the mCherry channel in red, mVenus in green and the overlay of cell trajectories in blue. (**B**) The ground truth or reference annotation of the “FUCCI-2channels” provided by the authors of the tool CellMAPtracer [19]. (**C**) Coordinates difference (in μm) between the FUCCItrack tracking and the true coordinates on the x and y axes based on the ground truth of CellMAPtracer (n = 8 cells during 61 frames each). The bars represent the mean difference, and the error bars the standard deviation.
(PDF)

S2 Fig Segmentation result of mCherry, mVenus and SiR-Actin channels. (**A**) Raw fluorescence images of mCherry, mVenus and SiR-Actin for a single cell. (**B**) Resulting binary masks after segmentation by FUCCItrack in white, with the contour of the cell and nucleus overlaid.
(PDF)

S3 Fig Cell and nuclear area increase over time. (**A**) Normalized cell and (**B**) nuclear area increase over time for individual daughter cells (n = 11 cells).
(PDF)

S4 Fig Nuclear area of an exemplary MCF7-FUCCI2 cell. (**A**) Fluorescence images (mCherry in red and mVenus in green) of the segmented nucleus of an exemplary MCF7-FUCCI2 cell. Scale bar is 100 μm. (**B**) Normalized nuclear area as a function of time for a MCF7-FUCCI2 cell.
(PDF)

S1 Movie Movie of a single MDA-FUCCI2 cell with its trajectory overlaid. (MP4)

S2 Movie Movie of a single MDA-FUCCI2 cell with a view centered on the cell over time. (MP4)

S3 Movie Movie of MCF7-FUCCI2 growing cluster over time. (MP4)

S4 Movie Movie showing the result of the segmentation of mCherry, mVenus and SiR-Actin channels for a single cell over time. (MP4)

S5 Movie Movie showing the result of the single cell tracking between a generation of cell. (MP4)
