# Supplementary material for: FUCCItrack: An all-in-one software for single cell tracking and cell cycle analysis
Source: PLoS One. 2022 Jul 6;17(7):e0268297. doi: 10.1371/journal.pone.0268297 (PMC9258891; doi:10.1371/journal.pone.0268297)
Supplement: S1 File — (PDF) [file pone.0268297.s001.pdf]

Supporting information for

FUCCItrack: an all-in-one software for single cell tracking and cell cycle analysis

Hubert M. Taïeb, Luca Bertinetti, Tom Robinson, Amaia Cipitria

Contents

FUCCItrack User's Guide 2
1. Introduction 3
2. Starting the program 3
3. Module description – Data loading 4
4. Module description – Collective 7
5. Module description – Single cell module 8
5.1 General components 9
5.2 Segmentation & tracking - Cell shape panel 13
5.3 Segmentation & tracking – Nucleus panel 15
6. Module description – Processing 16
6.1 Cell cycle analysis 17
6.1.1 mVenus panel 17
6.1.2 mCherry panel 17
6.1.3 Exporting the data 17
6.2 Migration analysis 18
6.3 Nucleus/Cell shape 19
7. Module description – Visualization & Saving 19
8. Module description – Concatenation 21
9. Panel description - Tools 23
10. Panel description - Status & Visualization 26
11. Saving FUCCItrack associated data and state 27

FUCCItrack User's Guide

1. Introduction

FUCCItrack is a 2D live cell visualization and analysis software. It allows users to analyze time lapse imaging data sets using the FUCCI/FUCCI2 systems, or any other two-color fluorophores. With the 2D time lapse data, FUCCItrack can perform numerous quantifications to study cell proliferation, migration, changes in nuclear and cell morphology as well as single cell cycle dynamics analysis. The graphical user interface (GUI) makes it easy for non-programming users to segment and track single cells in an automatic manner, while containing manual corrections functions to correct for errors.

Designed with the goal to be a multi-purpose platform, FUCCItrack is one software to visualize, analyze and create high quality visuals for presentation. It is modular by design and different features, or modules could be added to meet user specific needs. It is mainly automatic and therefore efficient in segmentation and tracking single cells, while being easy to use with an intuitive user interface and extensive documentation. FUCCItrack is designed to be open-source and hence has a licensing scheme that gives users the most freedom.

2. Starting the program

Select the FUCCItrack icon from the application menu in MATLAB or launch “FUCCItrack.exe” as a standalone software without MATLAB (for Windows users only). When FUCCItrack is running, a window like the one shown in Figure 1 appears on the screen. This starting page is divided in several major regions:

1) **Module toolbar:** provides quick access to some important modules. See specific module description.
2) **Tools:** allows user to navigate through the timelapse datasets and the single cell during segmentation and tracking.
3) **Status & Visualization** contains a log window to follow what is happening while using the software. It also contains a progress bar and button to create plots related to single cell tracking.

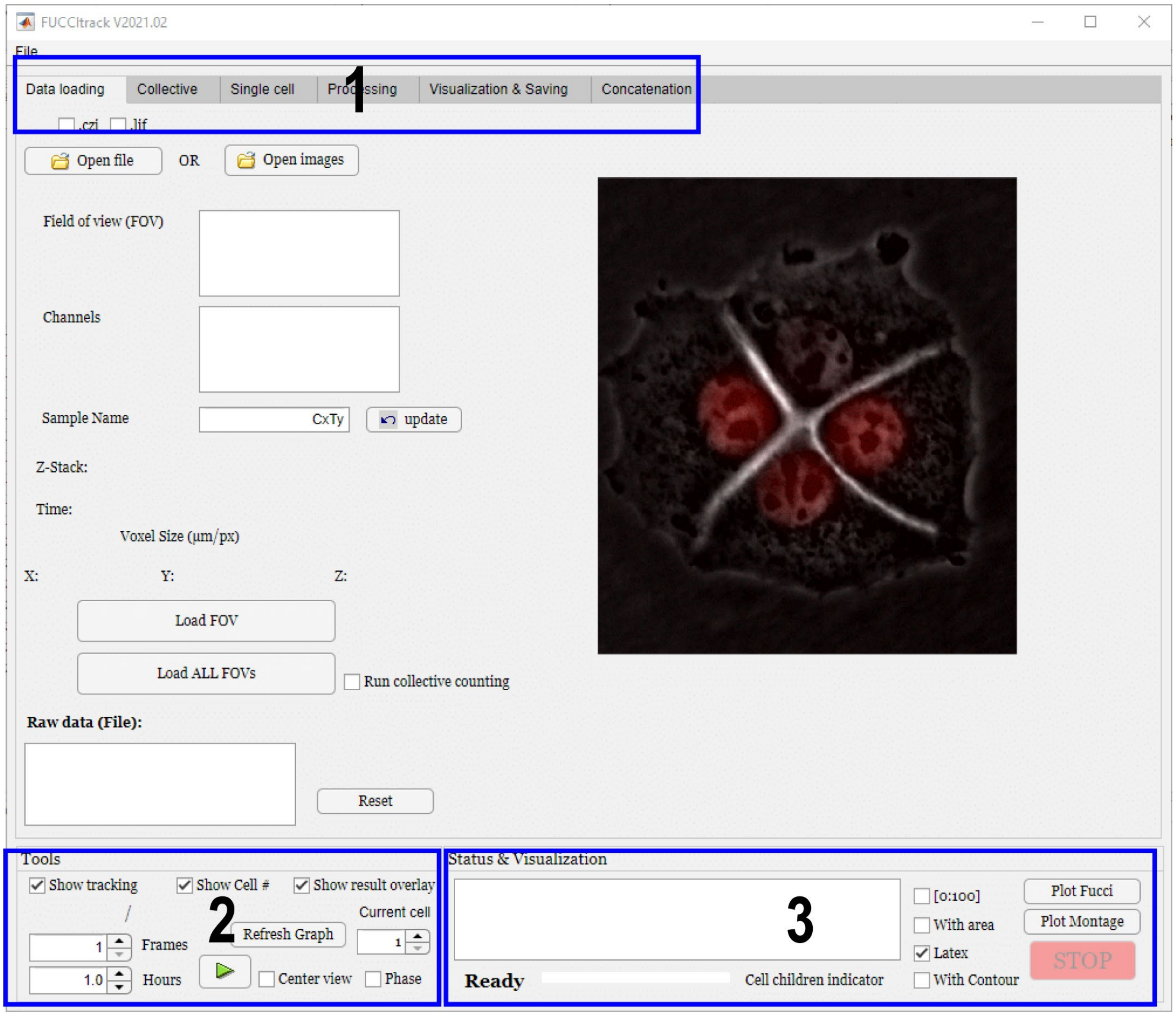

Figure 1. The FUCCItrack start page with three main regions: 1) the module toolbar, 2) the "Tools" panel and the 3) "Status & Visualization" panel.

3. Module description – Data loading

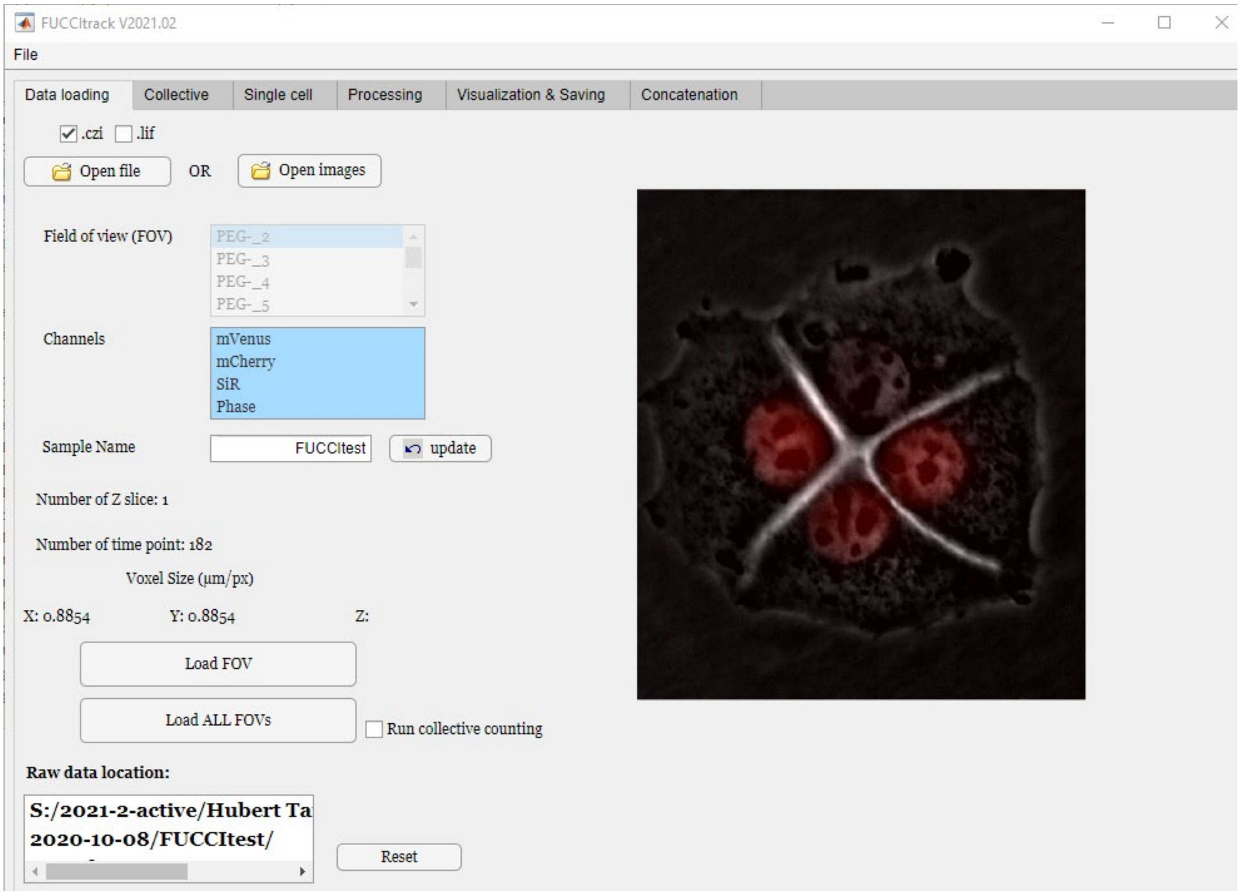

Figure 2. Data loading module

This is the first module that opens after launching FUCCItrack software. It contains all the different buttons and options to load the microscope data with the corresponding metadata. Please note that a minimum of two fluorescence channels (mCherry/mVenus) and one phase contrast or bright field or cell membrane dye (in our case approximated by live F-actin staining) are required for FUCCItrack.

.czi or .lif

User is invited to pick one of the two extensions presented corresponding to Zeiss or Leica microscope.

Open file

After picking the extension, user can press this button and select the location of the raw data file on the computer. This raw data can contain one or more fields of view (FOV).

Upon opening the files, metadata is read directly and updated in the software as presented below:

- "Field of view (FOV)": if the raw data contains several fields of view, it is possible to select the one of interest by directly clicking on the name. This is only possible with the "Open file" button since "Open images" can only load one field of view at the time.
- "Channels": this list contains the name of the channels used during the experiment and is updated automatically from the metadata of the raw files or the folder structure.

- “Sample name”: this field lets users choose the sample name corresponding to a specific FOV. All exported data and associated files from the software will start with the sample name as prefix. The “Update” button allows users to later change this sample name and update all files created within the software.
- “Number of Z slice”: this field is updated from the metadata and indicates the number of slices in the z stack. This value should always be 1 since 3D is not yet supported in the current version.
- “Number of time point”: this field enumerates the number of frames corresponding to number of time points in the time lapse experiment.
- “Voxel Size (μm/px)”: this field lists the voxel size in all directions and is updated from the metadata of the raw file. If the “Open images” button is selected, the user should manually input the voxel size.

Open images

This is to be used if the user does not have the raw data in the .czi or .lif format but rather a sequence of images corresponding to one field of view only. In this case, the images to be loaded should be with the .tif extension and organized in folders as follow:

- *Sample_name*/Raw data/mCherry/image_001.tif to image_182.tif
- *Sample_name*/Raw data/mVenus//image_001.tif to image_182.tif
- *Sample_name*/Raw data/Phase/image_001.tif to image_182.tif

With *Sample_name* replaced with the actual sample name, for example “FUCCItest” as seen above. Right after clicking on “Open images”, the user will be asked to select the folder that should be the one with the folder name corresponding to the sample name. The subfolder mCherry, mVenus and Phase have to be called like this.

If the raw data contains more than one FOV, then 2 options are possible.

Load FOV

User can pick the FOV of interest (by clicking on its name from the FOV list) and use the “Load FOV” button. The user will be asked to select a folder that will be used to store the processed data and results from this FOV (under a newly created folder named after the sample name). The software will then load all the images of this FOV inside the software, with no options to change the FOV afterwards.

Load ALL FOVs

This is intended for raw files that contains more than one FOV.

I. Without “Run collective counting”: this will load in batch all the FOVs from the raw data and for every FOV it will create a folder with the sample name containing three data files: “FUCCItest_tmp_data.mat” (includes all the data from the collective and single cell tracking), “FUCCItest_state.mat” (includes the state of every button of the software for future reloading of a session) and “FUCCItest_im_only.mat” (includes all the images of this FOV).
II. With “Run collective counting”: when this option is selected, the software will load in batch all the FOVs like previously but will also launch the collective module to directly obtain cell proliferation behavior. More details on the collective module are listed in the next section.

4. Module description – Collective

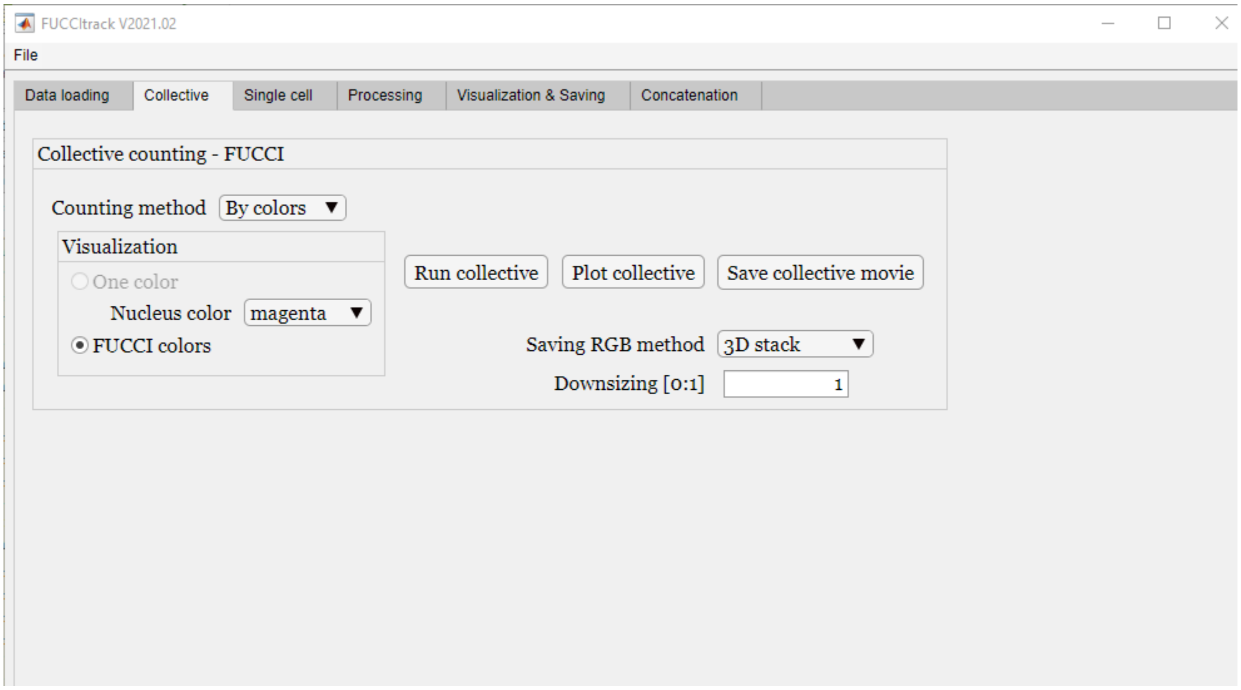

Figure 3. Collective module

The collective module allows user to run an automatic algorithm to segment and count the number of FUCCI/FUCCI2 cells over time.

Counting method

- “By colors”: this option enables the counting of the cell but with the information about which channel they belong. This is intended if user is interested in the fraction of mCherry^{+}/mVenus^{-} (G1 phase) and mVenus^{+} (S/G2/M phase).
- “Merged”: this option counts the cells independently of their channel of origin and gives a total number of cells as a function of time.

Visualization

Upon cell counting, one of the outputs is a time lapse video of the cells with their nuclei colored. There are 2 options as to what colors can appear on this video.

- “One color”: user can pick the colors they want the nuclei to be appearing.
- “FUCCI color”: the colors used will be the original FUCCI/FUCCI2 color, red and green.

Run collective

After selecting the options for the counting method and the visualization, the user can click this button to start the counting process.

Plot collective

This button will create a plot with the number of cells as a function of time, as well as the fraction of $mCherry^{+}/mVenus^{-}$ and $mVenus^{+}$ if the counting method “By colors” was chosen.

Saving RGB method

the software merges the phase and/or cell shape channel with the nuclei segmentation results and saves this RGB composite.

- “3D stack”: the output will be one multi-TIFFs file containing all the time points.
- “Sequential”: the output will consist in a sequence of tif files corresponding to every time point.
- “Downsizing [0:1]”: It is the ratio of the original size to reduced sized (in pixels). The user can pick a value between 0 and 1 and this will be used to downsize the original raw data for saving storage purposes.

Save collective movie

this button creates a movie from the raw images, overlaid with the nuclei segmentation. The color of the nuclei here is defined by the “Visualization” option.

5. Module description – Single cell module

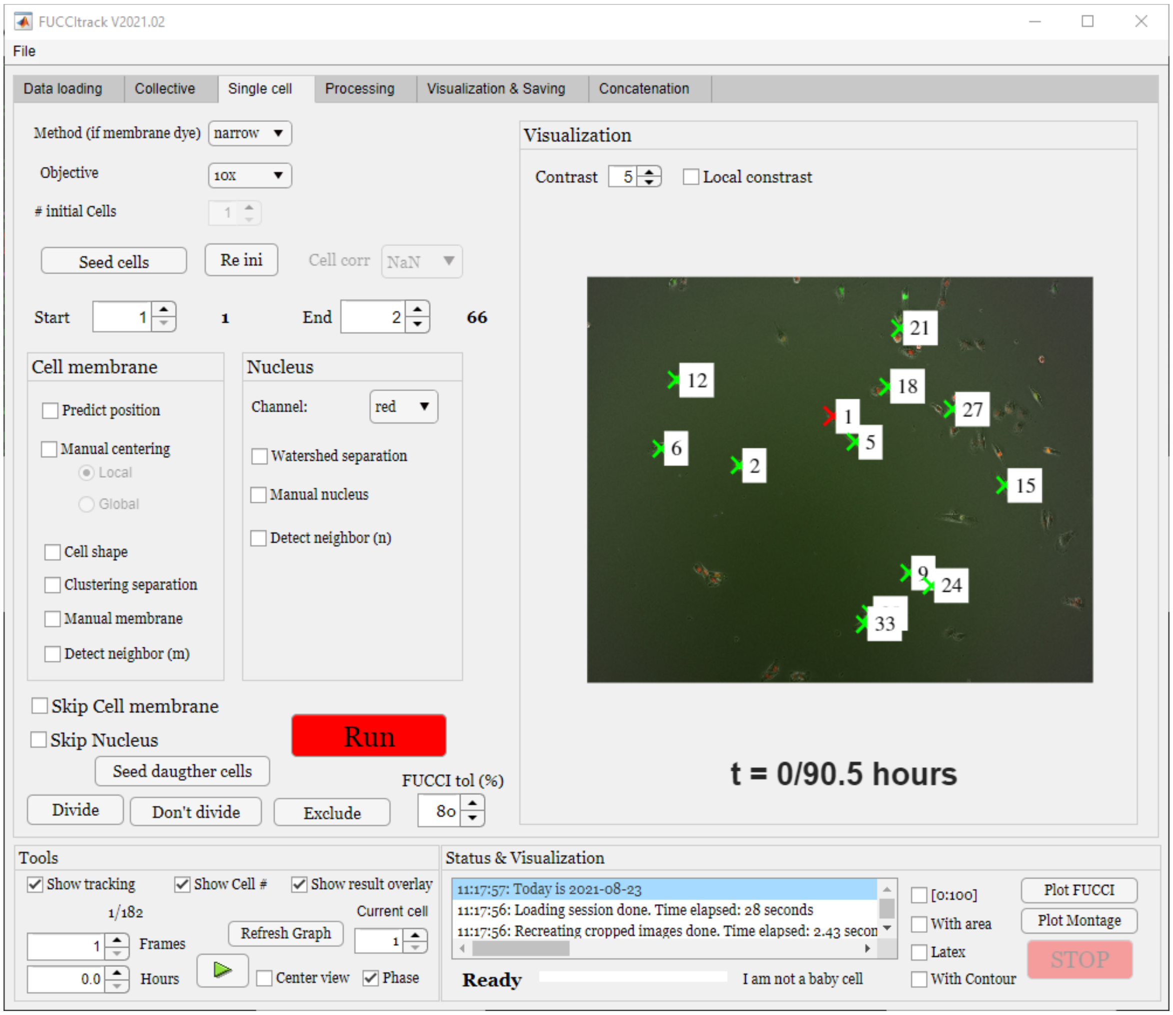

Figure 4. Single cell module with the “Tools” and “Status & Visualization” panels.

5.1 General components

This is the module to perform the segmentation and tracking of the single cells. In addition to the single cell module, the “Tools” and “Status & Visualization” panels are also shown since they are the most useful in this module.

During the segmentation and tracking, there is always a region of interest (ROI) that is defined around the cell of interest. The size of this ROI is defined as follows: cell diameter * margin, both being parameters of the software that can be tuned depending on preferences. The default values are 30 µm for the diameter and 2 for the margin, resulting in a ROI of 60x60 µm, to make sure the ROI captures at least one complete cell.

Method (if cell shape)

This button contains three possible options to perform the automatic cell segmentation (if a fluorescence dye is present). In all three methods, a local thresholding is performed to obtain a binary mask. The methods chosen will yield different threshold values for the segmentation.

- “Narrow”: this is the default method. It uses a ROI narrower than the initial ROI (60x60 µm) by 20% (this value can also be tuned by users in the source code) to calculate the threshold value. This is specifically useful when several cells are present in the ROI, resulting in a wrong threshold for the cell of interest.
- “Large”: this method uses the whole original ROI (60x60 µm) to compute the threshold value for the cell shape.
- “History”: this option is only available when at least one segmentation was done with the cell of interest. Instead of calculating a new threshold value every time point, the software will use the previously calculated threshold value to segment the cell in new time points. This method gives the best results with live stainings that are stable over time and for which a constant threshold value over time is enough to segment the cell shape.

If the user only has phase contrast or bright field, then this field has no impact. Indeed, there is no easy way to perform automatic segmentation on bright field of phase contrast imaging, so the user should only use the “Manual shape” button to manually segment the cell shape.

Objective

This field only has an informative value. Since retrieving this specific information from the metadata was not possible, the user can specify here the value of objective magnification used through this field.

# initial cells

The first time the dataset is loaded, it is possible to specify how many cells the user wants to select on the first time point. After clicking on the “Seed cells” button, the seeding procedure will be repeated in a row for as many cells as selected in the “# initial cells” field. It is also always possible to seed cells from a later point by clicking the “Seed cells” button in another frame.

Seed cells

This button will start the initialization procedure to select the cell of interest by opening a new window and a dialog box as shown in Fig. 5. The new window contains side by side the images coming from the different channels. First the user must zoom inside the field of view to better see the cell of interest (Fig. 5A). When the magnification is enough to see only the cell of interest, the user should click “Yes” on the dialog box that appeared asking “Done with zooming?” (Fig. 5B). The mouse cursor will change, and the user must click directly over the cell of interest. An initialization process will then start with this cell to segment the nucleus and cell whenever possible for the seeding time point (Fig. 5C).

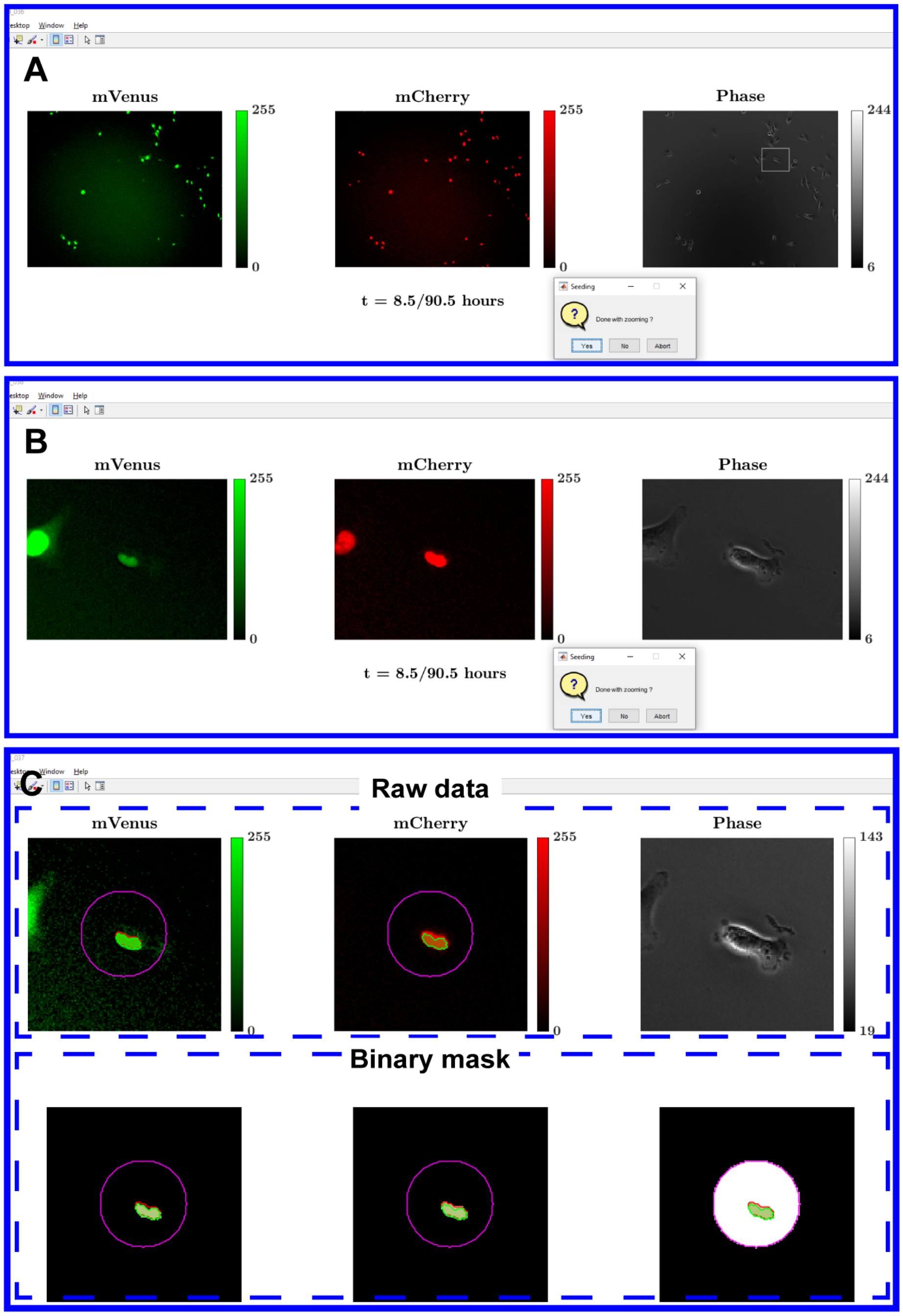

Figure 5. External window for single cell tracking and seeding cell procedure

Re ini

This field stands for “Rerun Initialization” and allows to start again the initialization phase with different settings in case the automatic segmentation failed.

Start and End

These two fields are used together with the “Run” button to specify for which time points the segmentation and tracking should be done. It ranges from 1 to the number of time points in the experiment. Next to these two fields are two numbers written in green that correspond to the beginning and end of the tracking for the current cell selected.

Skip cell shape

This option is only to be used if the segmentation and tracking was already performed and stored. It allows user to run again the segmentation of the nucleus, while keeping the cell tracking information stored. This can be useful if the automatic nucleus segmentation failed for example.

Skip nucleus

This option is an analogue to the previous function and let users run again the segmentation of the cell shape without modifying the cell nucleus segmentation.

Divide/Don’t divide/Exclude

These buttons work together and inform on the status of the current cell (Fig 6). For every tracked cell, a status concerning its cell cycle at the end of the tracking should be assigned by the user upon clicking on one of the three buttons. By default, all cells are considered excluded until a cell performs a division, or don’t divide by the end of the experiment. Exclusion criteria are defined by every user but can be (i) absence of mCherry or mVenus signal in the nucleus, (ii) cells getting out of the field of view during the tracking or (iii) failed division.

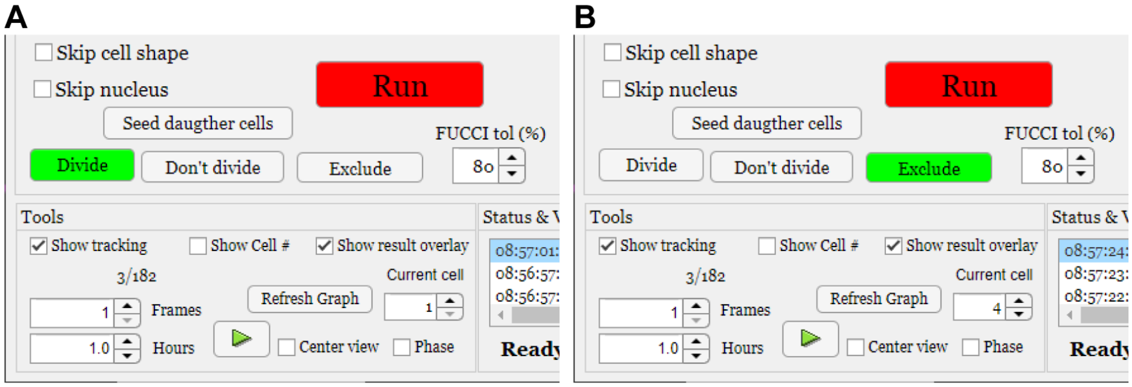

Figure 6. Divide/Don’t divide/Exclude example. **A)** User interface for a cell that divides and **B)** for a cell that is excluded.

This buttons only assign cells to a category within the software for the processing module. For example, only cells that divide will be used to calculate the duration of cell cycle.

Seed daughter cells

If the user is interested in tracking the daughter cells of a cell that divided, by pressing this button the seeding of the daughter cells will be done automatically with contrast to the “Seed cells” button that requires manual input. This button is equivalent to “Seed cells”, but does not require any inputs from the user since the last frame of the parent cells will be used and considered as the first frame of the two new daughter cells.

FUCCI tol (%)

In the ideal case scenario, the segmentation of the nucleus should be the same in shape and area for mCherry and mVenus channels. However, often the segmentation yields slightly different results for both channels. To address this discrepancy, this parameter (FUCCI tol, meaning FUCCI tolerance) is used to decide how much of the segmented nucleus in mCherry channel should overlap in the mVenus channel. The setting can be defined by the user and is set at 80 % by default. If the tolerance number is low, it means that segmentation in mCherry and mVenus could differ a lot, which will trigger a safety measure that will ask users to manually specify which one of the two channel yields the correct segmentation.

In terms of tracking and segmentation itself, certain options can be selected to assist the automatic tracking or let the user manually perform some tasks. These options are separated in “Cell shape” and “Nucleus” panel, as shown in Fig 7.

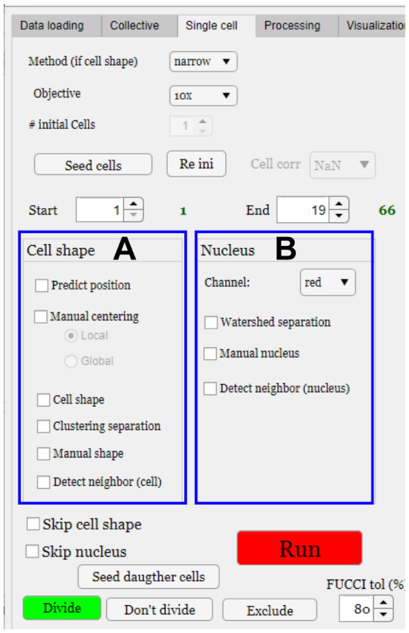

Figure 7. “Cell shape” and “Nucleus” panel

5.2 Segmentation & tracking - Cell shape panel

Please refer to Figure 7A to see where the options are in the GUI.

Predict position

After seeding, the program looks in the search window around the previous position for nuclei signal via automated adaptative thresholding. It then compares several features of the previous nucleus found with the potential candidates. The features used are: (i) area, (ii) distance to previous nuclei, (iii) eccentricity, (iv) mean intensity, (v) standard deviation intensity and (vi) maximum intensity. The software computes the relative difference between the previously correct position and the current candidates and looks for the minimum difference in every feature. The

cell chosen will be the one that has the most features with the minimum relative difference to the previous nuclei. If this option is not selected, the tracking starts from the previous position.

Manual centering

If this is selected, it forces the user to manually select the center of the nucleus in case other automated ways do not manage to localize the cell (due to nucleus signal too low, too many neighbor cells). Both the cell and nucleus will have to contain the position input by the user. It can be “local”, meaning the search window corresponds to the previous position found. But it can also be “global” in case the cell is out of the search window. In this case, the user can select the nucleus position from the total raw image.

Cell shape

By default, this option is not selected, and this means that FUCCItrack will not try to segment the cell shape. It will create an arbitrary circle around the center of the nucleus already previously found automatically or by user input. The circle is just a proxy for the cell outreach and its diameter can be modified by the user. If “Cell shape” is selected, the segmentation of the cell shape will be performed as shown in Figure 8.

Clustering separation (only relevant if cell shape is selected)

This is useful if you have two or ***k*** cells that are overlapping or touching. It allows to separate the cells using a ***k***-means clustering methods using the pixels from the segmentation as input for the clustering.

Manual shape (only relevant if cell shape is selected):

If the automated segmentation failed the user can manually draw the cell shape to perform the segmentation. This option is particularly necessary for users who whish to obtain cell shape based only on phase contrast or bright field since the automatic segmentation requires a fluorescence channel to perform well.

Detect neighbor (cell) (only relevant if cell shape is selected)

This option is useful if previously segmented cells are nearby the currently tracked cell. This option will delete previously segmented cells to avoid duplicate or wrong segmentation.

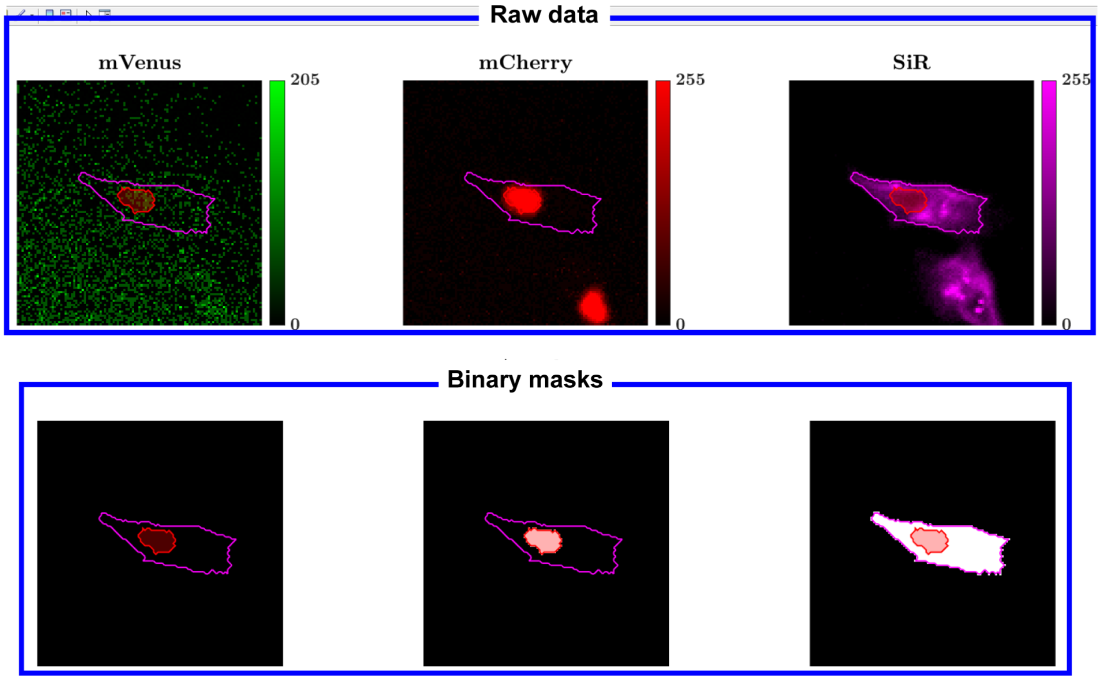

Figure 8. External window with cell shape segmentation

5.3 Segmentation & tracking – Nucleus panel

Please refer to Figure 7B to see where the options are in the GUI.

Channel

This option is to choose from which channel (red for mCherry and green for mVenus) the segmentation options described below should be applied to.

Watershed separation

If two nuclei are touching each other, FUCCItrack will run a watershed algorithm to separate them. The software picks the correct nucleus as the one which has its center of mass the closest to the cell position defined by the tracking.

Manual nucleus

This option allows the user to manually draw the nucleus in case the automatic segmentation failed.

Detect neighbor (nucleus)

This option works as "Detect neighbor (cell)". It will delete any cell nuclei previously found nearby the currently tracked cell, thus improving the automatic tracking performance.

6. Module description – Processing

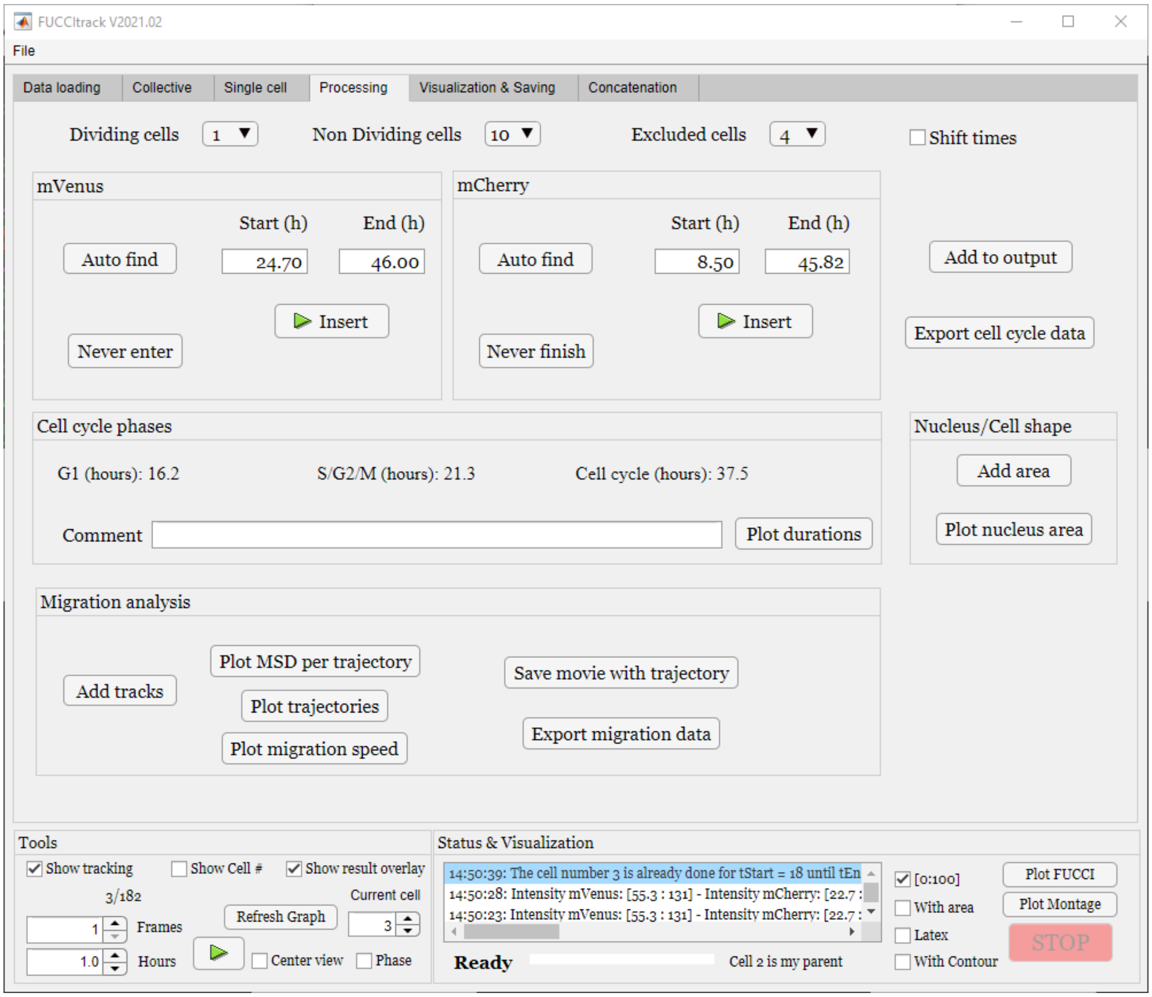

Figure 9. Processing module overview

The processing module allows user to compute and extract cell cycle phases, migration and nucleus/cell shape readouts. Users can go through the tracked cells using the “Current cell” in the Tools panel (see Fig. 12 and section 9 for more details). **It is important to not change the “Current cell” spinner while working on this module since all calculations are specific to the current cell.** Once all the readouts have been extracted for one cell, the users can change the “Current cell” spinner and procced with another cell.

<u>Dividing cells/ Non dividing cells/ Excluded cells</u>

This drop-down menu only summarizes the different status of cells tracked using the single cell module. Also, clicking on a cell number from this dropdown menu will have the same effect as using the “Current cell” spinner and will make the cell number selected the current cell.

<u>Shift times</u>

This option is used if you want to have the FUCCI/FUCCI2 intensity curves starting at 0 hour for every cell, even for the daughter cells that appeared during the experiment.

6.1 Cell cycle analysis

Both mCherry and mVenus panel have the same structure and are used to detect the beginning and end based on a threshold for the intensity defined as 5 % of the maximum intensity (this value can be modified by users).

6.1.1 mVenus panel

Auto find

This button will detect the time point when the mVenus curve goes above 5 % of its maximum intensity and assign the time stamp in the "Start (h)". By default, it will take the last time point of the tracking as the end for mVenus and assign the time stamp in the "End (h)" field.

Insert

After using the "Auto find", this field will stay red until pressed once. While red, it indicates that the value has not been saved in the application and users can still modify the field for the "Start (h)" and "End (h)" in case the "Auto find" failed. If the "Auto find" worked, the user will have to press on the button to save the data inside the application.

Never enter

This button can be pressed if cell stayed in G1 for the whole duration of the experiment and never had a signal for mVenus. The cell will not be excluded but considered as "non dividing cell". It means that the cell was in the category "G1 incomplete" and never started the S/G2/M phase. The field "Start (h)" will be set to the duration of the whole experiment and "End (h)" to 0.

6.1.2 mCherry panel

Auto find

This button works similarly as in mVenus with the exception that it detects the end of mCherry when the intensity drops below the 5 % threshold value and does not take the last time point. If the curve does not go below 5 % before division, the value for the end will be defined as the minimum of the mCherry curve, but only in the range after the start of mVenus.

Insert

This works exactly like previously described and should not be forgotten otherwise the value are not saved within FUCCItrack.

Never finish

This button can be pressed if the cell started the S/G2/M phase (by passing the 5 % mVenus threshold) but did not divide within the experiment. This category of cells is called "G2 incomplete".

6.1.3 Exporting the data

Comment

Users can write comments on the currently analyzed cell and this will be added to the exported file. For example: “Cell escapes field of view” etc...

Add to output

When the two “Insert” buttons and additional “Comment” have been added, the user can press this button to write the data into the output field that is exported.

Export cell cycle data

When the cell cycle analysis has been performed on all the tracked cells, the user can press this button to create the output files (one output file in .xlsx format and another in .mat format). The data present in this file is: cells and their parent identification number, beginning and end time of the mCherry and mVenus phase as well as the calculated t_{G1}, $t_{S/G2/M}$ and $t_{Cell\ cycle}$ in hours. It also contains the category of the cell (divided, did not divide, excluded) and the comments section that users may enter.

Plot durations

When more than one cell is tracked and the cell cycle analysis has been performed for all cells, the user can click on this button to create a boxplot with the duration of the different cell cycle phases (G1, S/G2/M and total division). The data corresponding to this plot can be seen after clicking the “Export cell cycle data” too.

6.2 Migration analysis

FUCCItrack uses the msdanalyzer package to store the migration related parameters such as trajectory, velocity and mean square displacement (MSD).

Add tracks

This button will use the tracking information and load this into the msdanalyzer class for every cell tracked. It is important to click on this button before any other in the “Migration analysis”. It also creates a “.mat” file that contains the class.

Plot MSD per trajectory

This button will compute and plot the mean square displacement (MSD) for every cell as a function of time.

Plot trajectories

This button will use the position data of every cell tracked and superpose their trajectories in a new figure.

Plot migration speed

This button will compute and plot the median of instantaneous speed over the trajectory of each cell in a boxplot format.

Save movie with trajectory

This button will create a movie containing the full trajectory of the cell (Supporting Movie 1).

Export migration data

This button will plot the other migration related metrics such as total distance, displacement and directionality in individual plots and export all of the data into an excel file.

6.3 Nucleus/Cell shape

This last panel can be used to compute and plot the nucleus projected area and the cell, if applicable.

Add area

This button will compute the nucleus area for every tracked cell and store the data inside the application.

Plot nucleus area

This button will create a new figure that contains the nuclear area as a function of time obtained by the "Add area" button previously.

7. Module description – Visualization & Saving

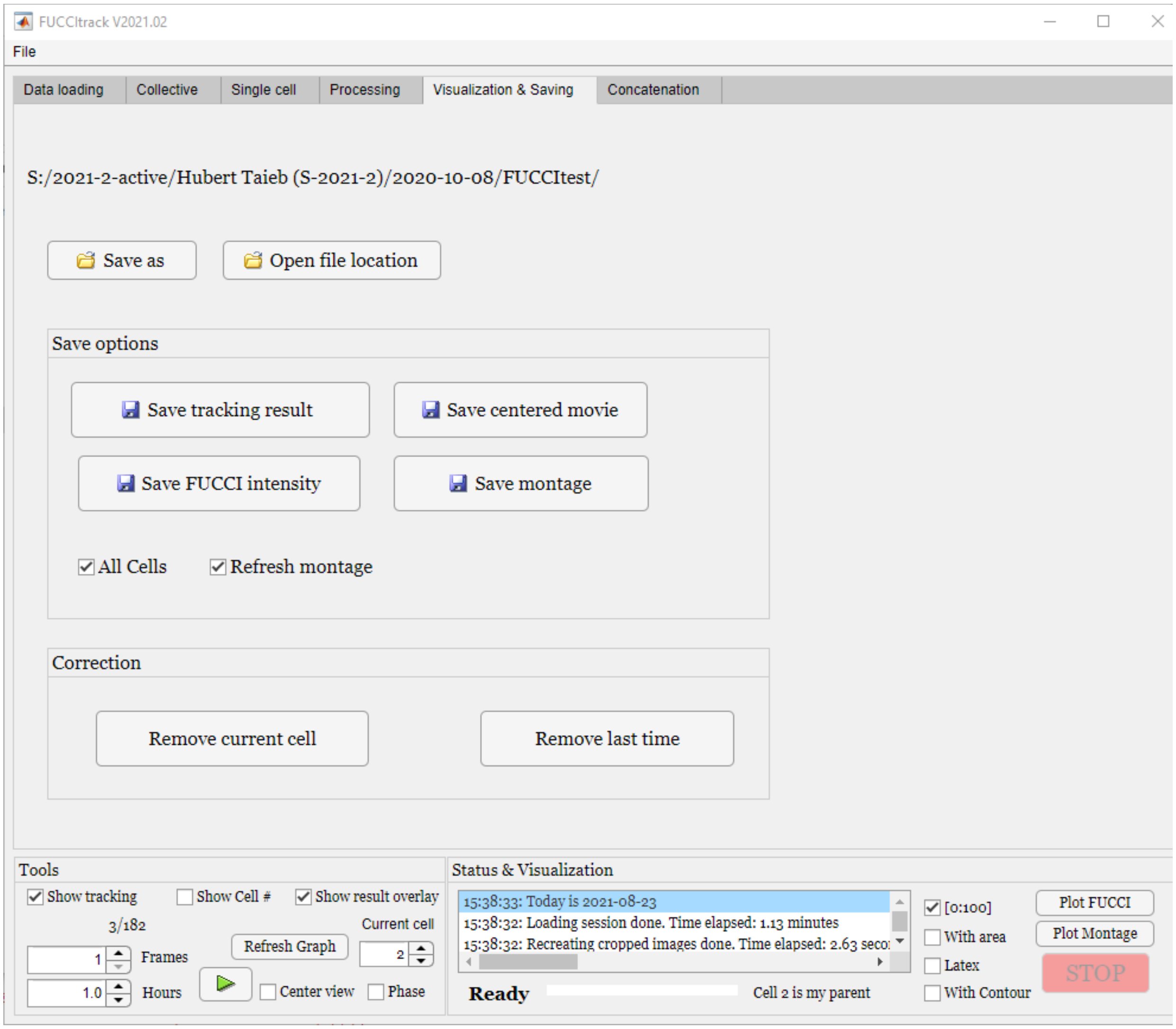

Figure 10. Visualization & Saving module

This part of the software (Fig. 10) is intended to be used to create a set of figures or movies concerning single cells.

Save as

The user is invited to select a location to save the FUCCItrack associated files ("tmp_data.mat", "im_only.mat" and "state.mat") to recreate the session in the future.

Open file location

This button opens the explorer where all the data related to this field of view is stored.

Save tracking result

This button takes a screenshot of Fig. 8 for every time point and saves this under the current cell specific folder.

Save centered movie

This button saves a movie that has the current cell centered over time during the tracking.

Save FUCCI intensity

This button will save the plots generated by “Plot FUCCI” in both pixel-based and vectorized format. In addition, the original MATLAB figure will be saved which contains the data of the intensity.

Save montage

This button will save the figure generated by the “Plot montage” button, also in both pixel-based and vectorized format. If the figure was already generated previously, then it will not recreate it, except if the “Refresh montage” option is selected.

- “Refresh montage”: this option will recreate the figure with the montage before saving it on to the computer.

All cells

If this option is selected, the saving of FUCCI intensity or montage will be done on all tracked cells instead of only the current cell.

Remove current cell

This button allows users to delete the current cell from the application data in case an error or a problem was associated with this cell.

Remove last time

This button will delete the data associated with the last time point of the currently tracked cell.

8. Module description – Concatenation

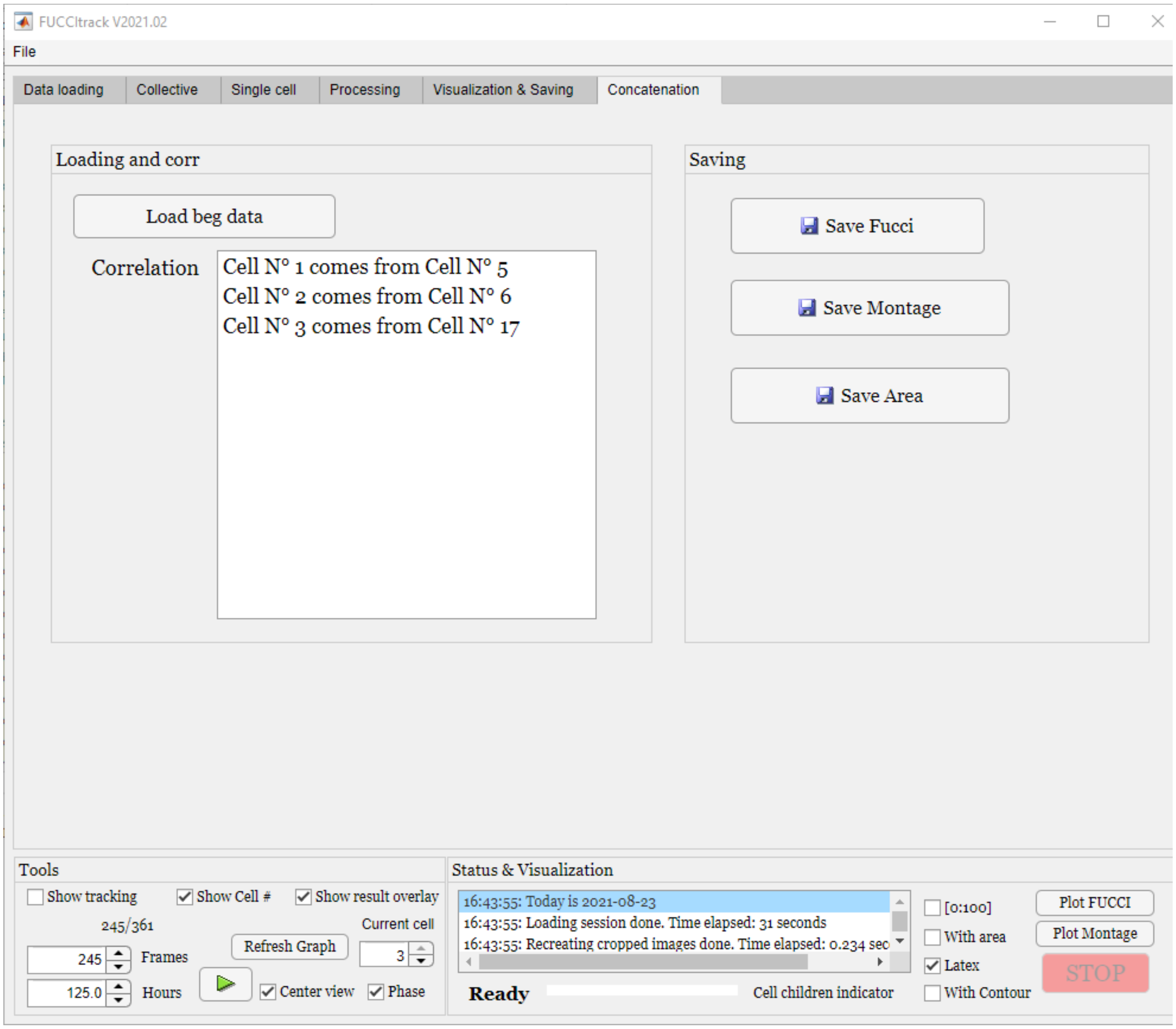

Figure 11. Concatenation module

The concatenation module (Fig. 11) is designed for users who have two set of files corresponding to the same field of view. The layout is simple and contains two main panels:

Load beg data

By clicking on this button, the data from the previous experiment will be loaded into the application.

Correlation

This is the part that links the two datasets. The line “Cell N°1 comes from Cell N°5” means that the current cell number 1 open in the current dataset is the same as the cell number 5 in the previous dataset. When the user clicks on the texts in this panel, the plots for FUCCI intensity, montage and area over time are generated.

Save FUCCI intensity

Like the processing module, this saves the FUCCI intensity with the two datasets concatenated.

Save montage

This button saves the montage across the two datasets.

Save area

This button saves the plot corresponding to the area over time from the two datasets.

9. Panel description - Tools

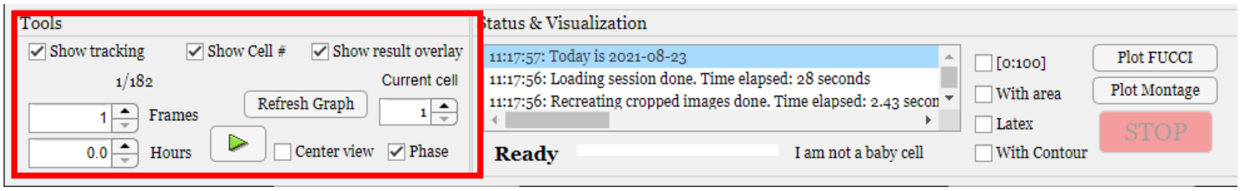

Figure 12. “Tools” and “Status & Visualization” panel

One important panel is the “Tools” panel, which is always present at the bottom of the app.

Show tracking

This option controls the visibility of the external window (Fig. 8). If the option is selected, the external window is visible, and hidden otherwise. Tracking and segmentation results process faster if this option is not selected at the cost that the user cannot follow in real time the tracking for eventual errors.

Show cell #

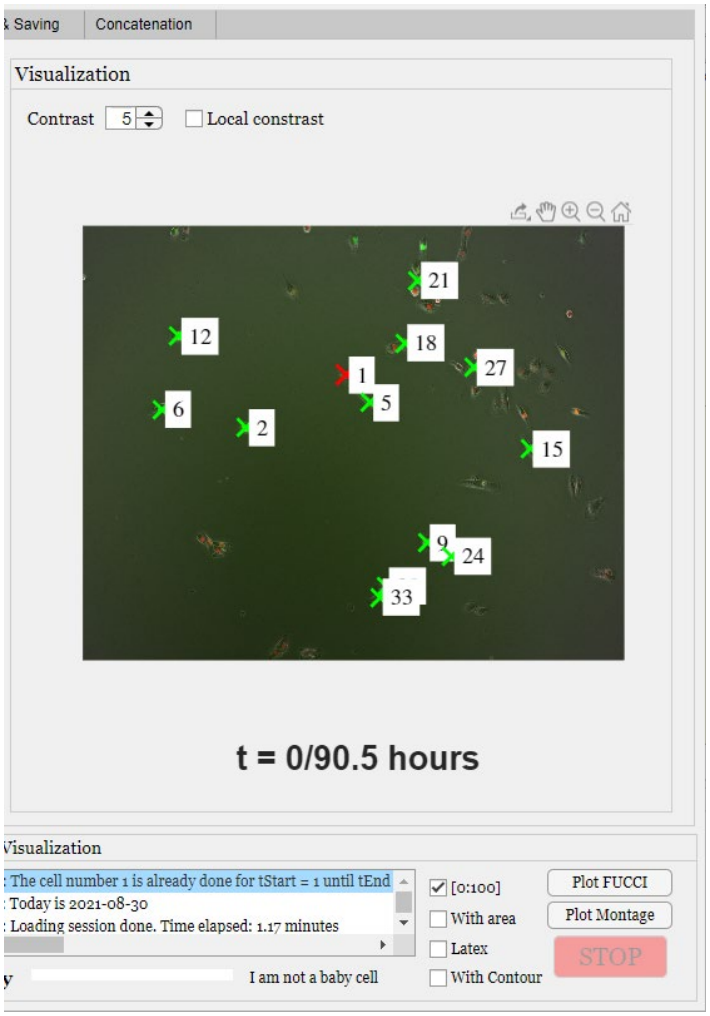

Figure 13. Internal visualization panel with all channels overlaid

This option will show or hide the cell number label on the internal “Visualization” panel (Fig. 13).

Show result overlay

This option will show or hide the result of the segmentation as an overlay on the raw and binary masks on the external viewer (Fig. 8, in magenta and red).

Current time point/total time point (1/182 here)

This will inform the user of the current time point and the total number of time points (here it is the first frame out of 182 time points).

Frames

This is a spinner and lets the user navigate through the time lapse by clicking on the up and down arrows. The user can also write a value directly on the text field on the left side.

Hours

This is a spinner connected to “Frames”. It translates the frame number to the actual time of experiment in hours. Users can also navigate through the time lapse by picking a specific hour or using the up and down arrows.

Play button

It will open the external window and show all time points that have been evaluated for the current cell, one by one until the end of the tracking.

Refresh graph

This button will open the external window for the current cell at its first time point. It is needed to have the external window open before using the “Run” button. The refresh graph can be useful if the current external window gets frozen during utilization by just recreating it.

Current cell

This spinner allows the user to go through the tracked cells and corresponds to the cell number or its unique identifier. It always starts at 1. While tracking a particular single cell, this spinner should always stay the same and user should not modify this during the tracking.

Center view

This option centers the internal visualization panel around the currently tracked cell instead of using the full field of view.

Phase

this option allows user to switch the internal and external viewers between phase and cell fluorescence channel if present (see Fig. 14).

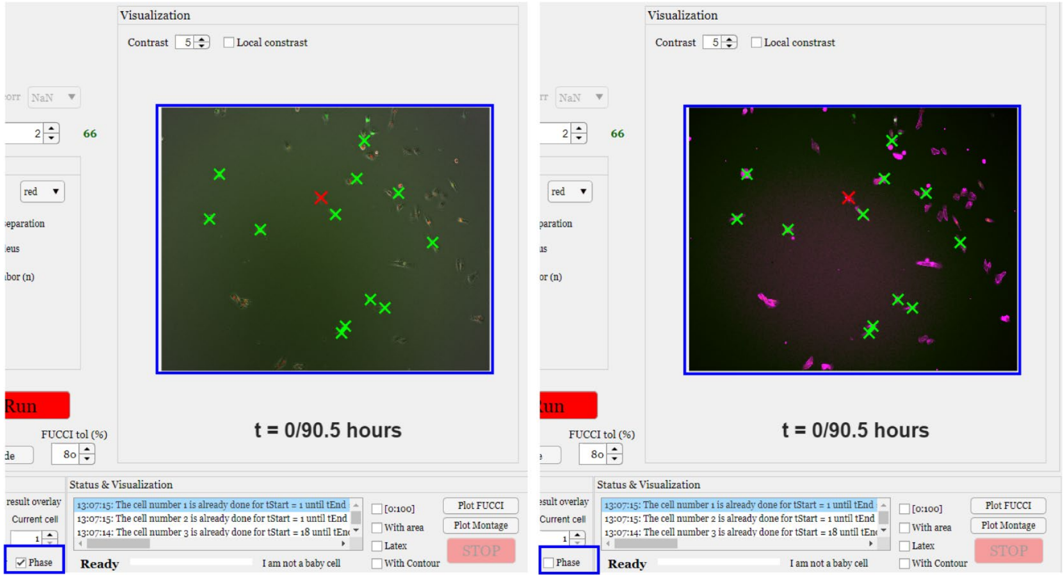

Figure 14. “Phase” option behavior**.** On the left panel, the “Phase” option is selected so that the phase contrast image is used for visualization. On the right panel, the “Phase” option is not selected, and the SiR-Actin channel is then visible.

10. Panel description - Status & Visualization

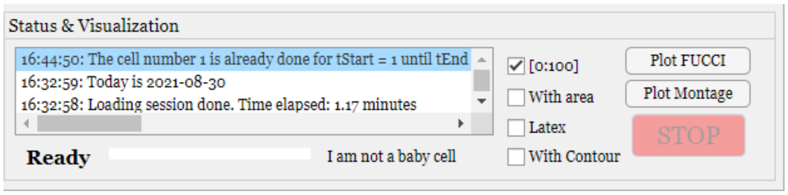

Figure 15. Status & Visualization panel

This panel contains a log field which is continuously updated (Fig. 15) . Every entry is recorded with its timestamp in the format “hh:mm:ss” to keep track of what has been done with FUCCItrack. There is also a progress bar (white at 0% and green when running) below the log field. Other fields related to status are:

Ready (or Busy)

This label field is an indicator that FUCCItrack is currently running operations in the background. When “Busy” is displayed in red, the user should wait and not click any button or perform operations.

“Cell children indicator” or “I am not a baby cell” or “Cell 1 is my parent”:

This label field change its text depending on the current cell selected. The label field will be updated upon tracking and gives user the information about the lineage of the current cell in the context of the single cell tracking.

- “Cell children indicator”: this is the default text when no single cell tracking data exists.
- “I am not a baby cell”: this will be displayed if the current cell was added by the “Seeding cells” button, with no information about its parent cell.
- “Cell XX is my parent”: this will be displayed (with the actual number XX depending on the experiment and current cells) when the current cell was added by the “Seed daughter cells” button, since the origin of the cell can be traced back to its parent.

The “Status & Visualization” panel also contains button and options to create two important plots: the FUCCI intensity and the montage of nucleus over time (Fig. 16).

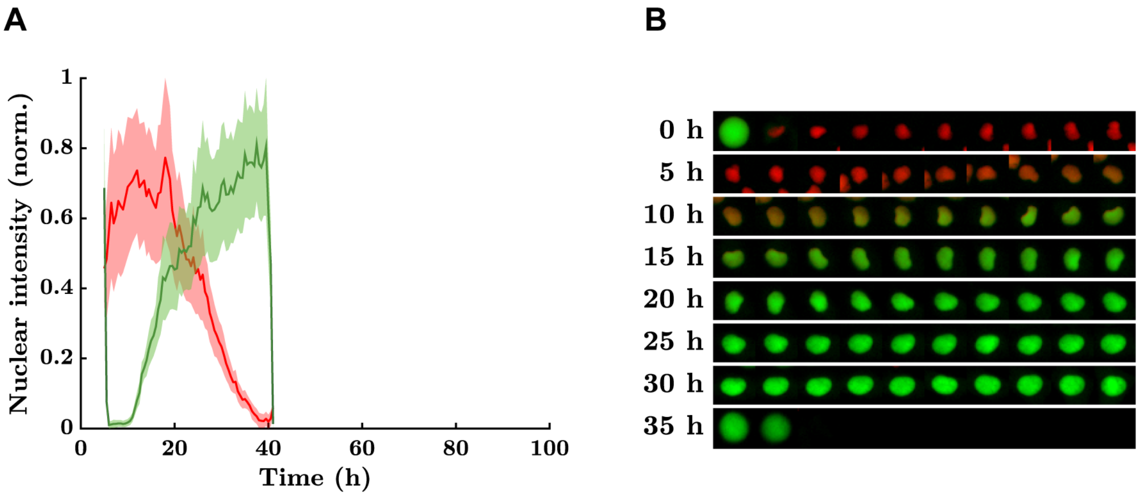

Figure 16. FUCCI intensity curves and corresponding nucleus montage.

<u>Plot FUCCI</u>

This button will create a new graph with the FUCCI intensity curves of the current cell (Fig. 16A). Two related options can be used before clicking on this button:

- "[0:100]": this defines the limits of the x axis (in hours) for visualization the FUCCI intensity curves. It is recommended to use this option if you want to compare FUCCI intensity curves belonging to different cells. If unselected, FUCCItrack will automatically determine the limit in hours corresponding to the current cell. The upper boundary can be modified by the user if necessary.
- "With area": when this option is selected, the FUCCI intensity plot will contain a second y axis on the right depicting the projected area of the current nucleus over time.
- "Latex": this option allows users to have latex style writing for all visualization plots. If not selected, the default font will be arial.

<u>Plot montage</u>

This button will create the figure corresponding of the concatenation of the nuclei of a cell over time (Fig. 16B). If the option "With contour" is not selected, then only the mCherry and mVenus channel will be visible. But if "With contour" is selected, the cell shape channel will also be present in the montage.

<u>STOP</u>

This button enables users to stop several functions of FUCCItrack while running. For example, if the tracking is started for 20 time points but it fails at an earlier time point, then user can press "STOP" and apply corrections.

11. Saving FUCCItrack associated data and state

FUCCItrack works with sessions that allows users to work on it, save it for later and reopen the application exactly where they left.

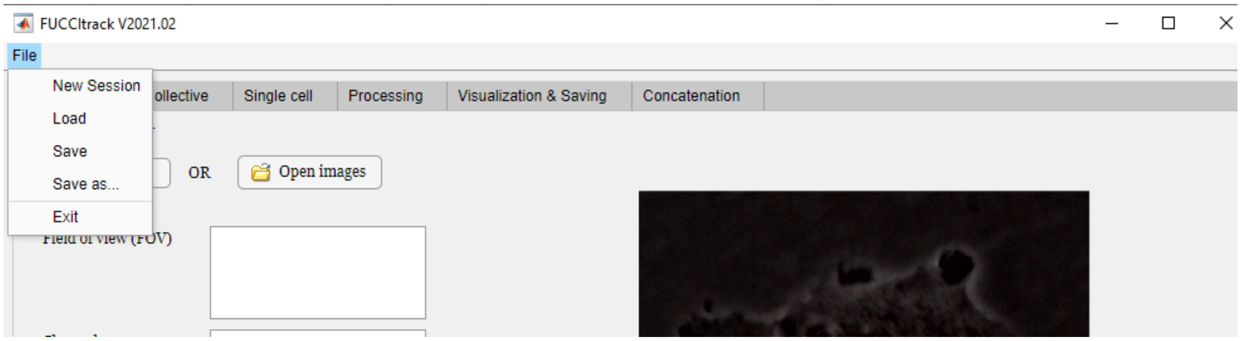

Fig 17. The File menu

New Session

This will reset the application to when the user opens it. Do not forget to use the "Save" button before clicking on this since all the data will be lost otherwise.

Load

The users will be invited to open the "tmp_data.mat" file of a previous session to load everything back to its saved state.

Save

This button will save the 3 associated MATLAB files ("tmp_data.mat", "im_only.mat" and "state.mat"), the files contain the necessary information for reloading the application as well as all the raw, tracking and segmentation data.

Save as ...

This button allows users to move all the MATLAB associated files to another location if needed.

Exit

This button terminates the software and closes it.
