# Supplementary figures and images for: FUCCItrack: An all-in-one software for single cell tracking and cell cycle analysis
Source: PLoS One. 2022 Jul 6;17(7):e0268297. doi: 10.1371/journal.pone.0268297 (PMC9258891; doi:10.1371/journal.pone.0268297)

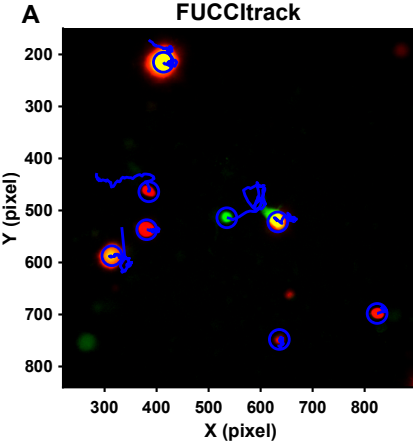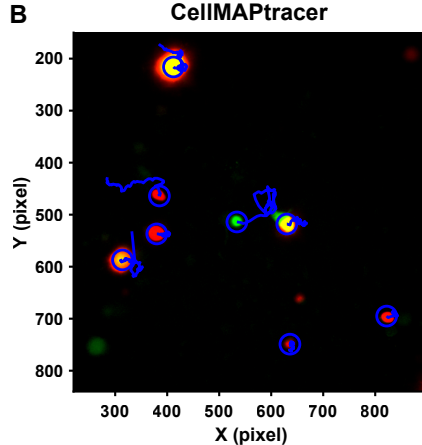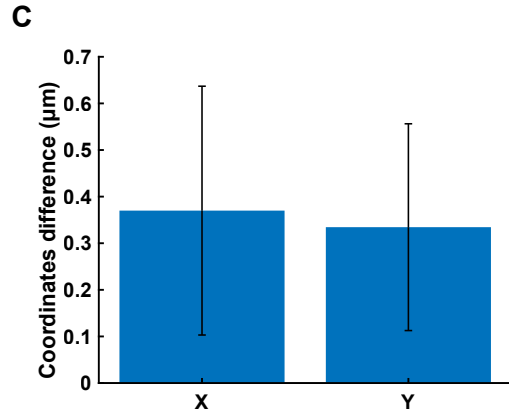

Supplement: S1 Fig — (A) Tracking results over the “FUCCI-2channels” datasets. The image contains the mCherry channel in red, mVenus in green and the overlay of cell trajectories in blue. (B) The ground truth or reference annotation of the “FUCCI-2channels” provided by the authors of the tool CellMAPtracer [19]. (C) Coordinates difference (in μm) between the FUCCItrack tracking and the true coordinates on the x and y axes based on the ground truth of CellMAPtracer (n = 8 cells during 61 frames each). The bars represent the mean difference, and the error bars the standard deviation. (PDF) [file pone.0268297.s002.pdf]

**A****Raw data**

mVenus

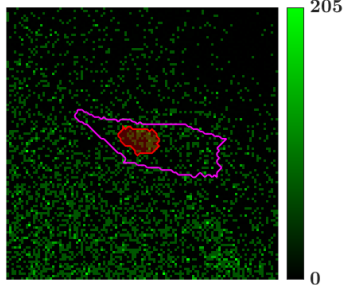

mCherry

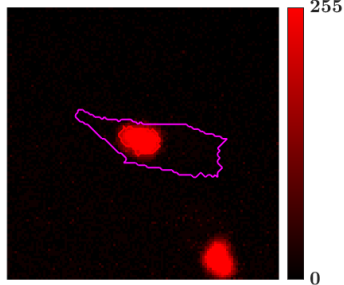

SiR

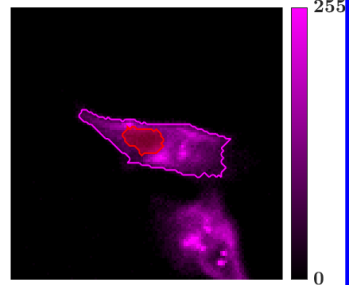**B****Binary masks**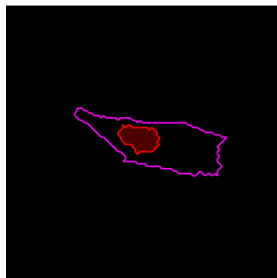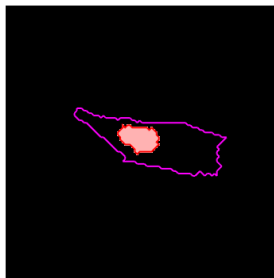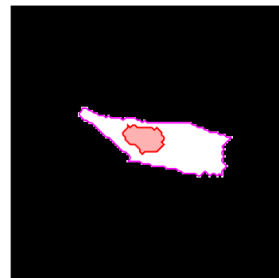

Supplement: S2 Fig — (A) Raw fluorescence images of mCherry, mVenus and SiR-Actin for a single cell. (B) Resulting binary masks after segmentation by FUCCItrack in white, with the contour of the cell and nucleus overlaid. (PDF) [file pone.0268297.s003.pdf]

**A**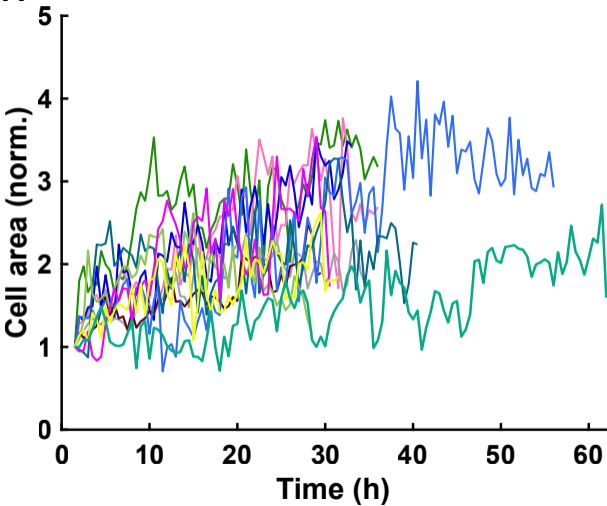**B**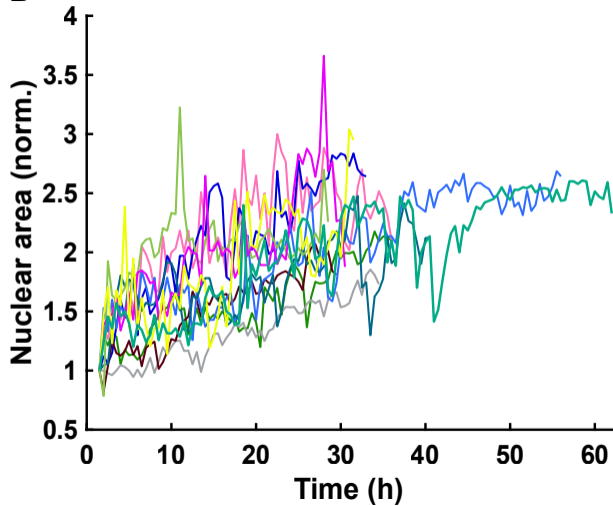

Supplement: S3 Fig — (A) Normalized cell and (B) nuclear area increase over time for individual daughter cells (n = 11 cells). (PDF) [file pone.0268297.s004.pdf]

**A**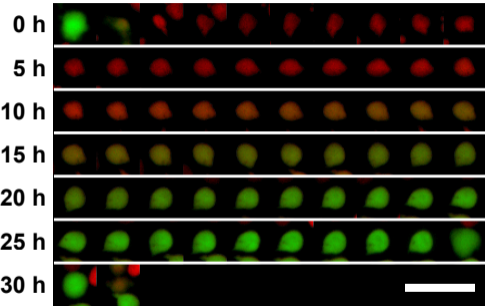**MCF7-FUCCI2****B**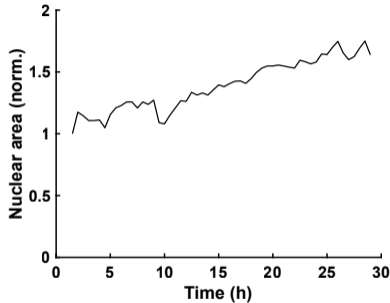

Supplement: S4 Fig — (A) Fluorescence images (mCherry in red and mVenus in green) of the segmented nucleus of an exemplary MCF7-FUCCI2 cell. Scale bar is 100 μm. (B) Normalized nuclear area as a function of time for a MCF7-FUCCI2 cell. (PDF) [file pone.0268297.s005.pdf]
